# Regulation of Adipogenesis and Key Adipogenic Gene Expression by 1, 25-Dihydroxyvitamin D in 3T3-L1 Cells

**DOI:** 10.1371/journal.pone.0126142

**Published:** 2015-06-01

**Authors:** Shuhan Ji, Matthew E. Doumit, Rodney A. Hill

**Affiliations:** Department of Animal and Veterinary Sciences, University of Idaho, Moscow, Idaho, United States of America; Complexo Hospitalario Universitario de Santiago, SPAIN

## Abstract

The functions of 1, 25-dihydroxyvitamin D (1, 25-(OH)_2_D_3_) in regulating adipogenesis, adipocyte differentiation and key adipogenic gene expression were studied in 3T3-L1 preadipocytes. Five concentrations (0.01, 0.1, 1, 10, 100nM) of 1, 25-(OH)_2_D_3_ were studied and lipid accumulation measured by Oil Red O staining and expression of adipogenic genes quantified using quantitative real-time PCR. Adipogenic responses to 1, 25-(OH)_2_D_3_ were determined on 6, and 12 h, and days 1-10 after induction of adipogenesis by a hormonal cocktail with or without 1, 25-(OH)_2_D_3_. In response to 1, 25-(OH)_2_D_3_ (1, 10, and 100 nM), lipid accumulation and the expression of *PPARγ*, *C/EBPα*, *FABP4* and *SCD-1* were inhibited through day 10, and vitamin D receptor expression was inhibited in the early time points. The greatest inhibitory effect was upon expression of *FABP4*. Expression of *SREBP-1c* was only affected on day 2. The lowest concentrations of 1, 25-(OH)_2_D_3_ tested did not affect adipocyte differentiation or adipogenic gene expression. The *C/EBPα* promoter activity response to 1, 25-(OH)_2_D_3_ was also tested, with no effect detected. These results indicate that 1, 25-(OH)_2_D_3_ inhibited adipogenesis via suppressing adipogenic-specific genes, and is invoked either during PPARγ activation or immediately up-stream thereof. Gene expression down-stream of PPARγ especially *FABP4* was strongly inhibited, and we suggest that the role of 1, 25-(OH)_2_D_3_ in regulating adipogenesis will be informed by further studies of adipogenic-specific gene promoter activity.

## Introduction

Growth of adipose tissue mass involves two distinct processes: hypertrophy (because of lipid synthesis and the subsequent increase in the size of adipocytes) and hyperplasia (because of proliferation, when preadipocyte and adipocyte numbers increase) [1]. Adipogenesis is the process of preadipocyte differentiation to form mature adipocytes, and during this process lipid accumulation occurs. The transcriptional control of adipocyte differentiation requires a sequential series of gene expression events and activation of a number of key signaling pathways [2]. This cascade starts with the induction of CCAAT/enhancer—binding protein β and δ (C/EBPβ and C/EBPδ). These two proteins then induce the expression of nuclear receptor peroxisome proliferator—activated receptor γ (PPARγ), which in turn induces *C/EBPα* expression [3]. Once expressed, C/EBPα activity positively feeds back on PPARγ activity. These two factors enhance each other’s expression and maintain the differentiated state [4]. Sterol-regulatory element binding protein 1c (*SREBP-1c*) is another notable key adipogenic gene [5]. *SREBP-1c* was independently discovered by two different research groups, and was named as ADD1 and SREBP-1c [6] [7]. This gene is induced during adipogenesis and is further regulated by insulin in cultured adipocytes [8,9]. In addition, SREBP-1c can modulate a variety of genes linked to fatty acid and triglyceride metabolism, and can also regulate adipogenesis [3] via induction of PPARγ gene expression through E box motifs present in the PPARγ promoter [10]. Increased expression of *SREBP-1c* leads to activation of *PPARγ* by inducing its expression and by increasing the production of an endogenous PPARγ ligand. All these transcriptional factors are necessary for the terminally differentiated phenotype.

Moreover, in humans, obesity is characterized by an increase in lipid accumulation and is the leading risk-factor for the development of Type 2 diabetes[11]. Understanding the biological process of adipogenesis is important for the development of novel targets for obesity therapy. Increasing evidence suggests there is a potential link between obesity and vitamin D insufficiency[12]. The bioactive metabolite of vitamin D is 1, 25 - (OH)_2_D_3_, which acts as a steroid hormone and a high-affinity ligand for the vitamin D receptor (VDR). The 1, 25 - (OH)_2_D_3_ activated VDR can form a heterodimer with the retinoid X receptor (RXR), which can bind to vitamin D response elements in various genes[13]. This VDR-RXR heterodimer may be competitive, inhibiting [14] the expression of PPARγ, which is a key regulator of adipogenesis, and thus also inhibit adipocyte maturation[13]. Therefore, 1, 25 - (OH)_2_D_3_ and VDR may play important roles in regulating adipogenesis. The vitamin D receptor is expressed very early in adipogenesis in 3T3-L1 cells. The VDR expression levels reach a maximum during the first 6 h after induction of differentiation, then decline to background levels after 2 days[15]. This creates a short window of opportunity for 1, 25 - (OH)_2_D_3_ to influence the differentiation process in forming mature adipocytes. Previous work has indicated that 1, 25 - (OH)_2_D_3_ is an inhibitor of adipogenesis in the 3T3-L1 cells[16,17]. In 1998, work performed by Kelly and Gimble [18] has established that 1, 25 - (OH)_2_D_3_ inhibits adipocyte differentiation in murine bone marrow cells. However, the specific mechanisms of the inhibitory actions of 1, 25 - (OH)_2_D_3_ in adipogenesis have not been described.

In the present study, we have determined the inhibitory effect of different concentrations of 1, 25 - (OH)_2_D_3_ in 3T3-L1 preadipocyte differentiation. We also studied the inhibitory activity of different concentrations of 1, 25 - (OH)_2_D_3_ on expression levels of key adipogenic genes (*C/EBPs* and *PPARγ*). As an important transcriptional factor during adipocyte differentiation, *C/EBPα* was a focus of the present study. We sought to determine whether there is a relationship between the inhibitory effect of 1, 25 - (OH)_2_D_3_ and the promoter activity of *C/EBPα*. Our study provides an experimental basis to better understand the function of 1, 25 - (OH)_2_D_3_ in regulation of adipogenesis, and the interactions between 1, 25 - (OH)_2_D_3_ and key adipogenic genes.

## Materials and Methods

### Materials

Mouse embryonic fibroblast cells (3T3-L1) were obtained from the American Type Culture Collection (ATCC). Dulbecco’s Modified Eagle’s Medium (DMEM), fetal bovine serum (FBS) and penicillin/streptomycin were from Gibco, Life Technologies (Grand Island, NY). Trizol, DNase I kit, high capacity cDNA reverse transcription kit (Cat # 4368814), and Taqman Master Mix were obtained from Life Technologies (Grand Island, NY). The Dual Reporter Luciferase Assay System was purchased from Promega Corporation, (Madison, WI). Oil Red O (ORO) powder, dexamethasone (D8893), insulin from bovine pancreas (I6634), 3-isobutyl-1-methylxanthine (IBMX) (I7018), and 1α,25-Dihydroxyvitamin D_3_ (D1530) were purchased from Sigma-Aldrich (St. Louis, MO). Mouse-specific anti-PPARγ (sc-7196) rabbit polyclonal antibody was purchased from Santa Cruz Biotechnology (Dallas, Texas). Mouse-specific anti-C/EBPα (ab139731) rabbit polyclonal antibody, and anti-β-actin mouse-monoclonal (ab8226) were purchased from Abcam (Cambridge, MA). AlexaFluor 680 anti-rabbit IgG was from life Technologies (Grand Island, NY) and IRDye800 anti-mouse IgG was from Li-Cor (Lincoln, NE).

### Cell culture

Mouse 3T3-L1 preadipocytes were cultured at 37°C with 5% CO_2_ enriched air in DMEM with 10% FBS, 100 I.U. /ml penicillin, 100 μg/ml streptomycin (basal growth medium). Cells were seeded in 6-well plates and 24-well plates with glass cover slips in basal growth medium and cultured until confluent. On day 0 (two days post confluence), 1, 25-dihydroxyvitamin D was added to the differentiation medium at the following final concentrations: 100, 10, 1, 0.1, and 0.01 nM, and cultures were incubated for 48 h. Cells grown in basal growth medium without 1, 25-dihydroxyvitamin D served, as a negative control. Cells grown in medium containing basal growth medium with dexamethasone (1 μM), IBMX (500 μM) and insulin (1.7 mM) (standard hormonal differentiation medium, DM) served, as a positive control. For the DMI treatment, insulin, dexamethasone and IBMX were provided for the first 48 h followed by only insulin in basal growth medium throughout the remaining time points. Media were changed every 2 days for all treatments. Cells were harvested on 0, 6, and 12 h, and days 1, 2, 4, 6, 8 and 10 for RNA extraction, or protein extraction. Parallel cultures were stained with ORO and representative images of ORO stained cells on day 10 were quantified using MetaMorph Image analysis software (Nashville, TN).

### Cells and transfection

For each cell culture well, 3.5×10^5^ 3T3-L1 cells were plated and allowed to reach 80% confluence. Cells were then co-transfected with 2 μg [pGL4.10 (luc2/-500 C/EBPα)] and 0.2 μg of internal transfection control vector [pGL4.74 (hRluc/TK)], and transferred to growth medium. Cells were incubated 24 h, and allowed to reach 100% confluence. Two days post confluence cells (0 h) were treated with 100 nM of 1, 25(OH)_2_D_3_ plus differentiation medium, differentiation medium only, or growth medium only. Cells were harvested on 0, 12, 24, and 24 h, and assayed for firefly luciferase and renilla luciferase activities using the Dual Reporter Luciferase Assay System (Promega, Madison, WI) and a Wallac 1420 Multi Label Counter. Firefly luciferase activity units were normalized to units of renilla luciferase activity to correct for transfection efficiency.

### Oil Red O (ORO) and Hematoxylin staining

Accumulation of lipids was observed using ORO staining[19]. Oil Red O in isopropanol stock solution (3.5 mg/ml) was prepared, stirred overnight and filtered. Cells grown on cover slips in 24-well plates were used for lipid staining. On the day of the time point, culture medium was removed and cells were gently rinsed once with phosphate buffer saline (PBS). Cells were fixed in 10% formaldehyde in PBS for 1 hr at RT. After fixation, cells were rinsed with PBS and then 60% isopropanol. Oil Red O solution (6:4 v/v of stock solution and water) was added and incubated for 10 min at RT. Finally, cells were washed with distilled water, three times. Hematoxylin counter staining was done according to the manufacturer’s instructions. Briefly, cells were incubated with filtered hematoxylin for two minutes and rinsed twice with tap water. Differentiation solution (0.25% HCl in 70% alcohol) was added and cells were rinsed again with tap water. The glass cover slips were then removed from the wells and inverted on to a glass slide with mounting medium (Vecta Shield, Vector Labs, Burlingame, CA).

### RNA extraction and cDNA synthesis

Total RNA was extracted using Trizol according to the manufacturer’s instructions. The RNA pellet was resuspended in nuclease-free water and stored at -80°C until further use. RNA was quantified using a Nanodrop ND-1000 UV-Vis Spectrophotometer (Nanodrop Technologies, Wilmington, DE). The quality of RNA was verified on 1% agarose gels. Two μg of RNA from each treatment was DNase treated. Synthesis of cDNA was conducted using a high capacity cDNA reverse transcription kit and random hexamers as primers according to the manufacturer’s instructions. To ensure availability of cDNA sufficient to perform all real-time PCR reactions, cDNA synthesized from 2 μg of RNA was pooled for each sample. Pooled cDNA was diluted 1:10 using nuclease-free water for real-time PCR.

### Real-time PCR

Quantitative real-time PCR was performed using Taqman MGB primer/probe sets with an ABI 7500 Fast Real Time PCR system (Applied Biosystems, Foster City, CA). Primers and probes for all genes were designed using Applied Biosystems Primer Express 3.0 software. Primers (Integrated DNA Technologies, Coralville, IA) and probes (Life Technologies, Grand Island, NY) were designed to have a Tm of 58–60°C and 69–70°C, respectively. Primer-probe sets that span exon-junctions (trans-intronic positions) were chosen for real-time PCR, to prevent binding to genomic DNA (Table 1). Eukaryotic translation elongation factor 2 (*EEF2*) was used as an endogenous control for gene expression. Probes were labeled with 6-FAM or VIC for all target genes or control (*EEF2*), respectively. Real-time PCR assays for each sample were conducted in duplicate wells with all genes including endogenous control on the same plate. Reactions contained Taqman Universal Fast PCR Master Mix, No AmpErase UNG (Applied Biosystems, Foster City, CA) (1X), forward primer (0.5 μM), reverse primer (0.5 μM), Taqman probe (0.125 μM) and cDNA template made up to a final volume of 15 μl in nuclease-free water. Real-time PCR cycle conditions included a holding time of 90°C for 20 sec, followed by 40 cycles of 90°C for 3 sec and 60°C for 30 sec of melting and extension temperatures, respectively.

**Table 1:** Primer-probe sets for real-time PCR.

*Accession No*. */ Gene name*	*Sequences*
***NM_007907*.*1 /*** *Eukaryotic translation elongation factor 2 (Eef2)*	*FP*: *CTGCCTGTCAATGAGTCCTTTG*
	*RP*: *GCCGCCGGTGTTGGAT*
	*Probe*: *CTTCACCGCTGATCTG*
***NM_011146*.*2 /*** *Peroxisome proliferator activated receptor gamma (PPARγ)*	*FP*: *GCTTCCACTATGGAGTTCATGCT*
	*RP*: *AATCGGATGGTTCTTCGGAAA*
	*Probe*: *TGAAGGATGCAAGGGTT*
***NM_007678*.*3 /*** *CCAAT/enhancer binding protein alpha (C/EBPα)*	*FP*: *CGCAAGAGCCGAGATAAAGC*
	*RP*: *GTCAACTCCAGCACCTTCTGTTG*
	*Probe*: *AACGCAACGTGGAGAC*
***NM_009504*.*4*** *Vitamin D receptor (VDR)*	*FP*: *GGCTTCCACTTCAACGCTATG*
	*RP*: *TGCTCCGCCTGAAGAAACC*
	*Probe*: *CCTGTGAAGGCTGCAA*
***NM_009883*.*3 /*** *CCAAT/enhancer binding protein beta (C/EBPβ)*	*FP*: *GCGCACCGGGTTTCG*
	*RP*: *GCGCTCAGCCACGTTTG*
	*Probe*: *ACTTGATGCAATCCGGA*
***NM_007679*.*4 /*** *CCAAT/enhancer binding protein delta (C/EBPδ)*	*FP*: *CTGTGCCACGACGAACTCTTC*
	*RP*: *GCCGGCCGCTTTGTG*
	*Probe*: *CGACCTCTTCAACAGC*
***NM_024406*.*1 /*** *Fatty acid binding protein 4 (FABP4)*	*FP*: *CCGCAGACGACAGGAAGGT*
	*RP*: *AGGGCCCCGCCATCT*
	*Probe*: *AAGAGCATCATAACCC*
***NM_010052*.*3 /*** *Preadipocyte factor-1 (Pref-1)*	*FP*: *AATAGACGTTCGGGCTTGCA*
	*RP*: *GGTCCACGCAAGTTCCATTG*
	*Probe*: *CTCAACCCCCTGCGC*
***NM_011480*.*3 /*** *Sterol regulatory element binding transcription factor 1 (Srebf1)*	*FP*: *GCGGTTGGCACAGAGCTT*
	*RP*: *CTGTGGCCTCATGTAGGAATACC*
	*Probe*: *CGGCCTGCTATGAGG*
***NM_009127*.*4 /*** *Stearoyl-Coenzyme A desaturase 1 (Scd1)*	*FP*: *CAACACCATGGCGTTCCA*
	*RP*: *TGGGCGCGGTGATCTC*
	*Probe*: *AATGACGTGTACGAATGG*

Primers and probe sequences used in real-time PCR listed 5’ to 3’: Forward primer (FP), reverse primer (RP) and Taqman probes for the following genes were designed from the corresponding GenBank accession numbers.

Data were analyzed using the relative C_T_ (∆∆Ct) method[20]. Average Ct values of endogenous control (*EEF2*) were subtracted from target gene average Ct values of each gene, to obtain ∆Ct values of each gene for each sample. For each gene, ∆Ct values in the control treatment at each time point were used to normalize ∆Ct values of corresponding time points of each treatment to obtain ∆∆Ct and mRNA fold expression values [2^(-∆∆Ct)^].

### Western Blot

Protein extraction from 3T3-L1 preadipocytes and adipocytes was performed using cell lysis buffer with addition of phosphatase and protease inhibitors (Cell Signaling Technologies, Danvers, MA). The supernatant was extracted by centrifugation and protein concentration was determined by BCA protein assay according to manufacturer’s protocol (Thermo Scientific, Rockfrod, IL). Ten μg of whole cell lysate was resolved on SDS-PAGE (4–10% precise gels) and then transferred to PVDF membrane. Membranes were blocked with 5% non-fat milk in 1X TBST for 1h at room temperature. Membranes were incubated with anti-PPARγ, anti-C/EBPα and anti-β-actin at 4°C overnight, then washed with 1X TBST and incubated with AlexaFluor 680 conjugated anti-rabbit IgG and IRDye 800 conjugated anti-mouse IgG for 1 h at room temperature. After thorough washing, blots were scanned and quantified using an Odyssey Dual Infrared Imaging System (Li-Cor).

### Microscopy

Images were obtained using a Nikon 80i phase-contrast microscope, using a 20X objective lens. Image quantification was performed using MetaMorph Image analysis software (Nashville, TN). Area fractions were collected for each image. Images were collected in six to eight replicates from each culture well. Average area fractions of each of the six to eight replicates were used to calculate average area fraction of each treatment sample. Further, average area fraction values of each treatment were normalized to average area fraction values of corresponding controls.

### Statistical analysis

Statistical analysis software (SAS) 9.3 was used to perform all data analysis. Data were analyzed using a one-way analysis of variance (ANOVA) for each time point. Tukey’s test was used to find the significant differences among the different means. Differences, when *p* < 0.05, were considered statistically significant. Gene expression data were analyzed by comparing log (base 2) transformed values of mRNA fold expression across treatments within each time-point. All data are reported as mean ± SE (n = 3).

## Results

### 1, 25 - (OH)_2_D_3_ inhibits lipid accumulation

**Fig 1:**
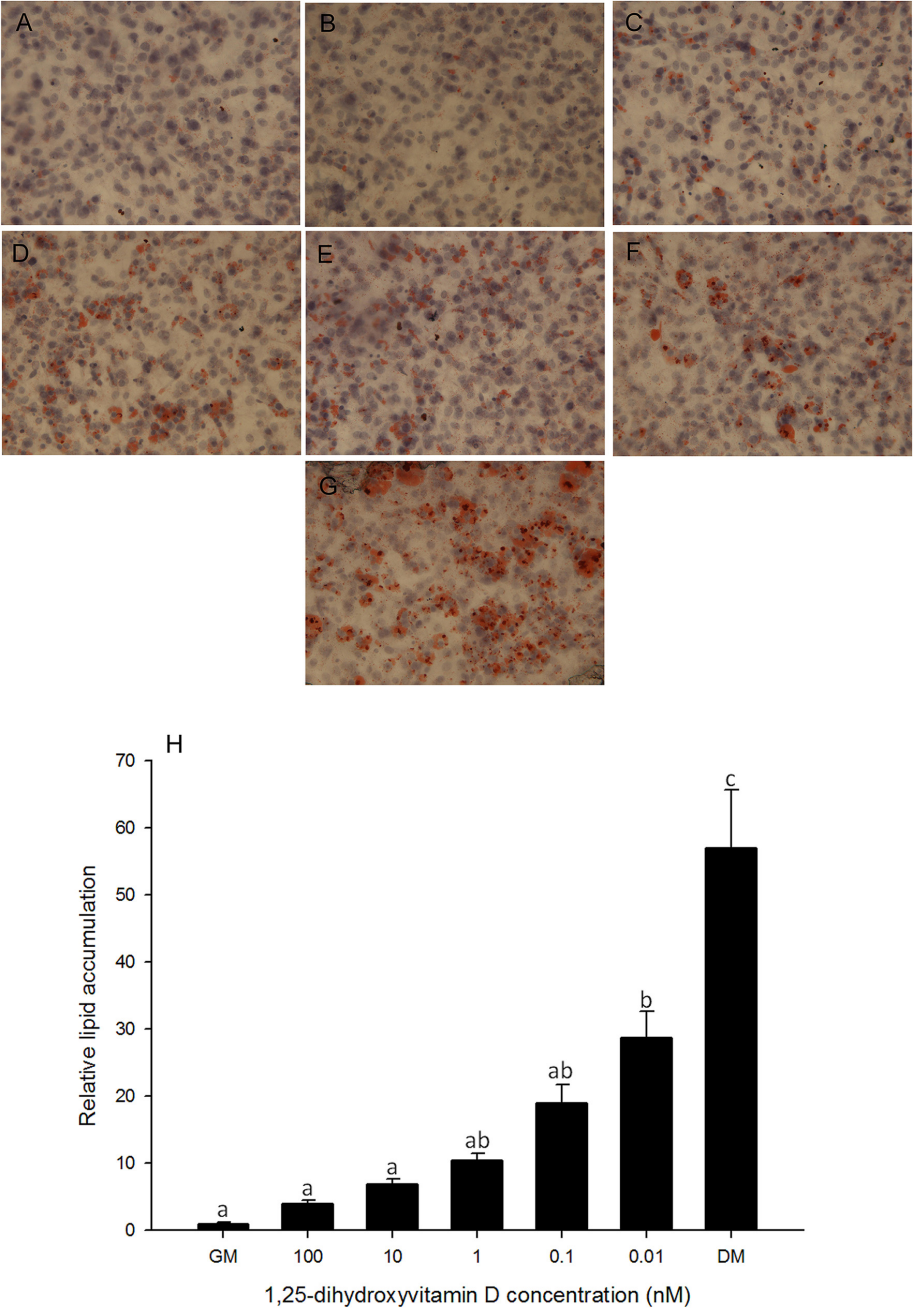
Oil Red O staining in 3T3-L1 cells. Cells were treated with basal growth medium (GM) (A) or differentiation medium plus different concentrations of 1, 25 - (OH)_2_D_3_, 100 nM (B), 10 nM (C), 1 nM (D), 0.1 nM (E) or 0.01 nM (F) or differentiation medium (DM) (G). Oil Red O staining was performed on days 2, 4, 6, 8 and 10. Representative day 10 images are shown. Images were collected at 400x magnification. (H): Quantification of lipid accumulation in 3T3-L1 cells. Lipid accumulation was quantified using MetaMorph Image analysis software. Area fractions were collected for each treatment and normalized to control of corresponding time point. Data are means ± SE (n = 3). Different letters represent treatment effects that were significantly different (*P* < 0.05). The dose-response effect of 1, 25 - (OH)_2_D_3_ treatment on lipid accumulation is illustrated.

Cultures of 3T3-L1 cells were incubated in standard hormonal differentiation medium, in the presence or absence of 1, 25 - (OH)_2_D_3_. DMI medium served as a positive control treatment. Basal growth medium served as a negative control. Lipid accumulation was observed through ORO staining on days 0, 2, 4, 6, 8 (data not shown) and 10 (Fig 1A). Image quantification analysis shows that lipid accumulation at higher concentrations of 1, 25 - (OH)_2_D_3_ (100, 10, 1 nM) treated cells was similar to that in negative control cells, and significantly lower than the positive control (Fig 1B). The lowest concentration of 1, 25 - (OH)_2_D_3_ treated cells showed higher lipid accumulation than negative control and other 1, 25 - (OH)_2_D_3_ treated cells, however, lipid accumulation was still significantly lower when compared to the positive control (Fig 1B). This suggests that 1, 25 - (OH)_2_D_3_ treatment inhibited lipid accumulation and adipogenesis in a dose dependent manner.

### High concentrations of 1, 25 - (OH)_2_D_3_ inhibit PPARγ expression

**Fig 2:**
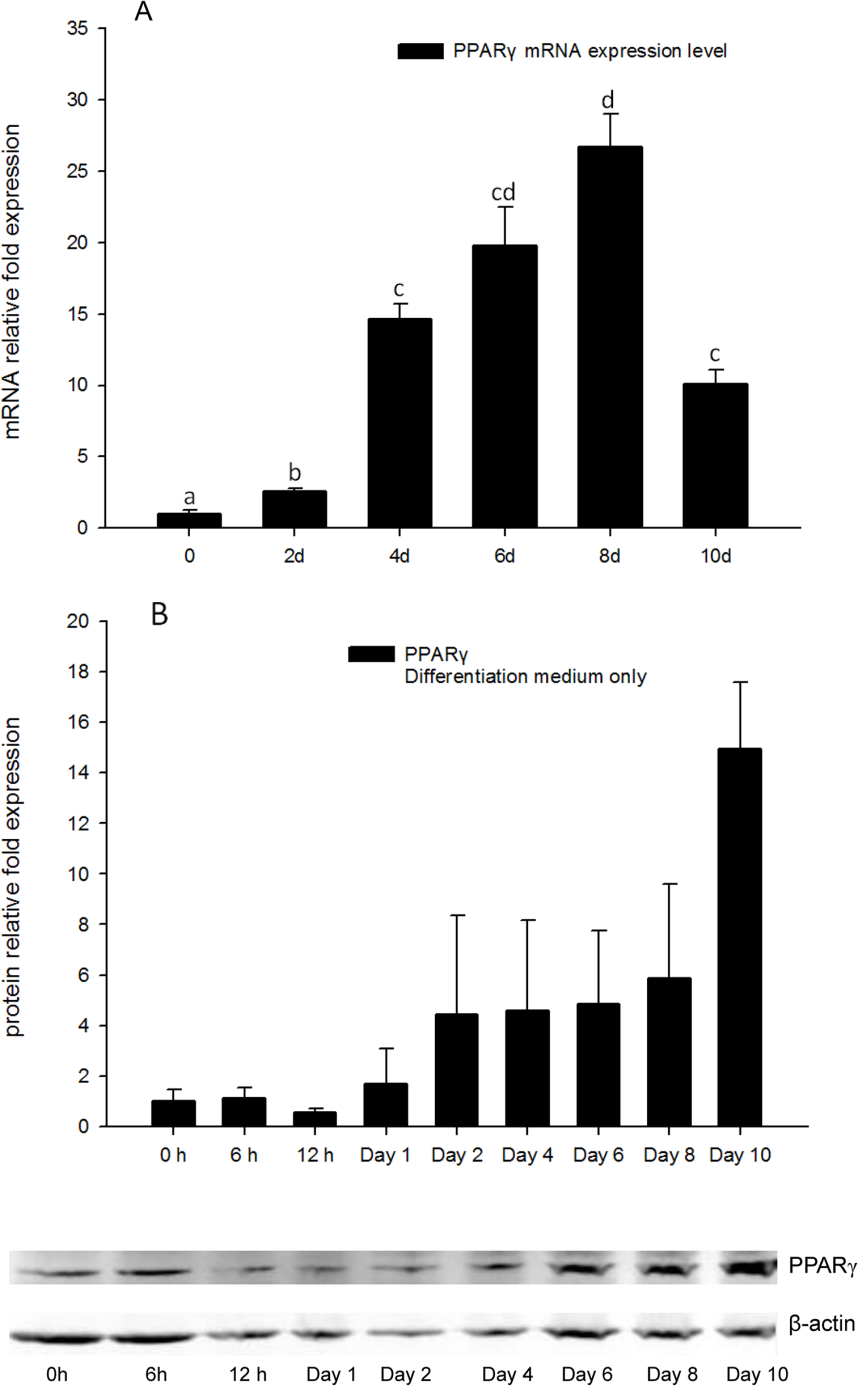
(A) Real-time PCR quantification of *PPARγ* gene expression in DM treatment of 3T3-L1 cells on days 0, 2, 4, 6, 8, and 10 with *EEF2* used as endogenous control (∆Ct). Data were normalized to *PPARγ* gene expression of the day 0 group (∆∆Ct). (B). Image showing Western blot analysis (Odyssey Dual Infrared Imaging System (Li-Cor)) of PPARγ on 0, 6, and 12 h, days 1, 2, 4, 6, 8 and 10. β-actin was used as an internal protein loading control. Quantification of PPARγ normalized to β-actin is shown. Data are means ± SE (n = 3). Different letters represent treatment effects that were significantly different (*P* < 0.05).

To better understand the expression pattern of *PPARγ* during the process of adipocyte differentiation, RNA extracts and protein extracts from 3T3-L1 cells treated with DM only were obtained for real-time PCR and Western blots tests. Gene expression levels of *PPARγ* began to increase after day 2, and reached a maximum on day 8 (Fig 2A). Protein levels of PPARγ were consistent with gene expression levels, increasing on day 2, and reaching the highest level on day 10 (Fig 2B). This suggests *PPARγ* expression level increased concurrent with adipocyte differentiation, and consistent with increasing lipid accumulation.

**Fig 3:**
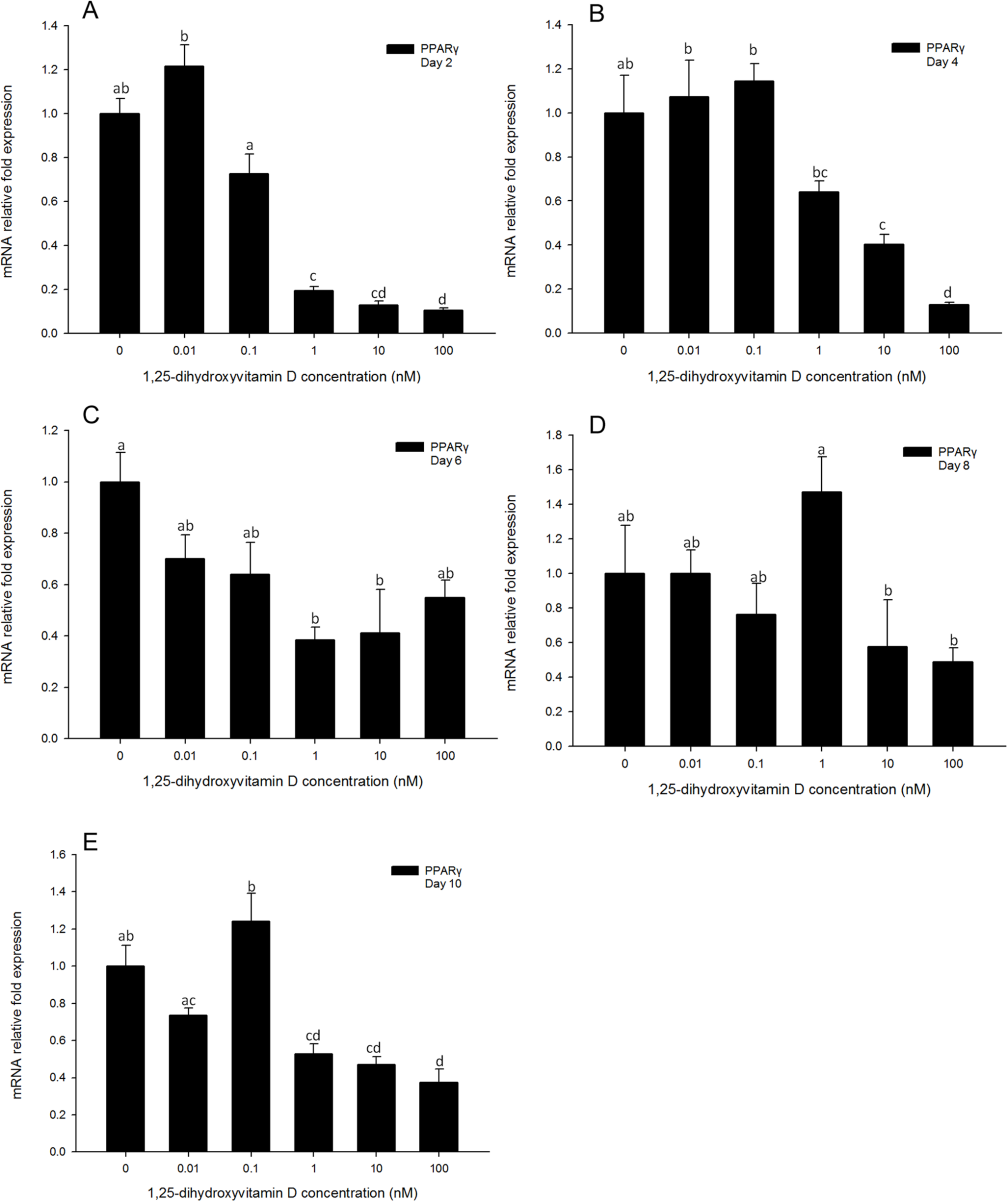
Real-time PCR quantification of *PPARγ* gene expression in 3T3-L1 cells on days 2 (A), 4 (B), 6 (C), 8 (D) and 10 (E). Cells were treated with DM in the presence or absence of 0.01, 0.1, 1, 10, and 100 nM 1, 25 - (OH)_2_D_3_ and *EEF2* was used as endogenous control (∆Ct). Data were normalized to *PPARγ* gene expression of the positive control (DM) at the corresponding time point (∆∆Ct). Data are means ± SE (n = 3). Different letters represent treatment effects that were significantly different (*P* < 0.05).

Gene expression levels of *PPARγ* in 3T3-L1 cells treated with high concentrations (100, 10, and 1 nM) of 1, 25 - (OH)_2_D_3_ were significantly inhibited compared to the positive control (Fig 3A–3E) at all time-points measured. In addition, for all time-points, 3T3-L1 cultures treated with low concentrations (0.1 and 0.01 nM) of 1, 25 - (OH)_2_D_3_ showed no significant differences in *PPARγ* gene expression levels as compared to positive control cultures (Fig 3A–3E). On days 2, 4 and 10, the highest concentration (100 nM) of 1, 25 - (OH)_2_D_3_ had the greatest inhibitory effect on *PPARγ* mRNA expression levels. This suggests that *PPARγ* gene expression levels were inhibited by 1, 25 - (OH)_2_D_3_, and 1, 25 - (OH)_2_D_3_ had greater efficacy in inhibiting *PPARγ* gene expression at higher concentrations.

To confirm the real-time PCR results, Western blots also were performed on whole cell lysates following treatment with 100 or 1 nM of 1, 25 - (OH)_2_D_3_ at all the time points. Basal growth medium (GM) and differentiation medium (DM) served as negative and positive controls, respectively. PPARγ protein levels were inhibited by 1, 25 - (OH)_2_D_3_ at 6 h (S1A Fig), but thereafter the PPARγ protein levels were high in the 100 nM 1, 25 - (OH)_2_D_3_ treated groups, but remained low in DM only groups from 12 h to day 4 (S1B–S1E Fig). On day 8, both the 1, 25 - (OH)_2_D_3_ treated groups and DM only group had high PPARγ protein level (S1G Fig), and on day 10, PPARγ protein levels in both 100 and 1 nM of 1, 25 - (OH)_2_D_3_ treatment groups decreased to levels similar to the GM only treatment. However, in the DM only group, PPARγ protein level still remained relatively high (S1H Fig). This suggests that 1, 25 - (OH)_2_D_3_ treatments inhibit PPARγ protein levels only at the early time point, 6 h, and again at the late time point, day 10. At the other time points, since the whole cell lysates were used for Western blots measurement, and the 1, 25 - (OH)_2_D_3_ treated group had higher PPARγ protein level than the DM only group, suggesting that the inhibitory efficacy of 1, 25 - (OH)_2_D_3_ on adipogenesis may function at the level of blocking PPARγ protein trafficking to nucleus.

### Gene expression of C/EBPα is inhibited by high concentrations of 1, 25 - (OH)_2_D_3_

**Fig 4:**
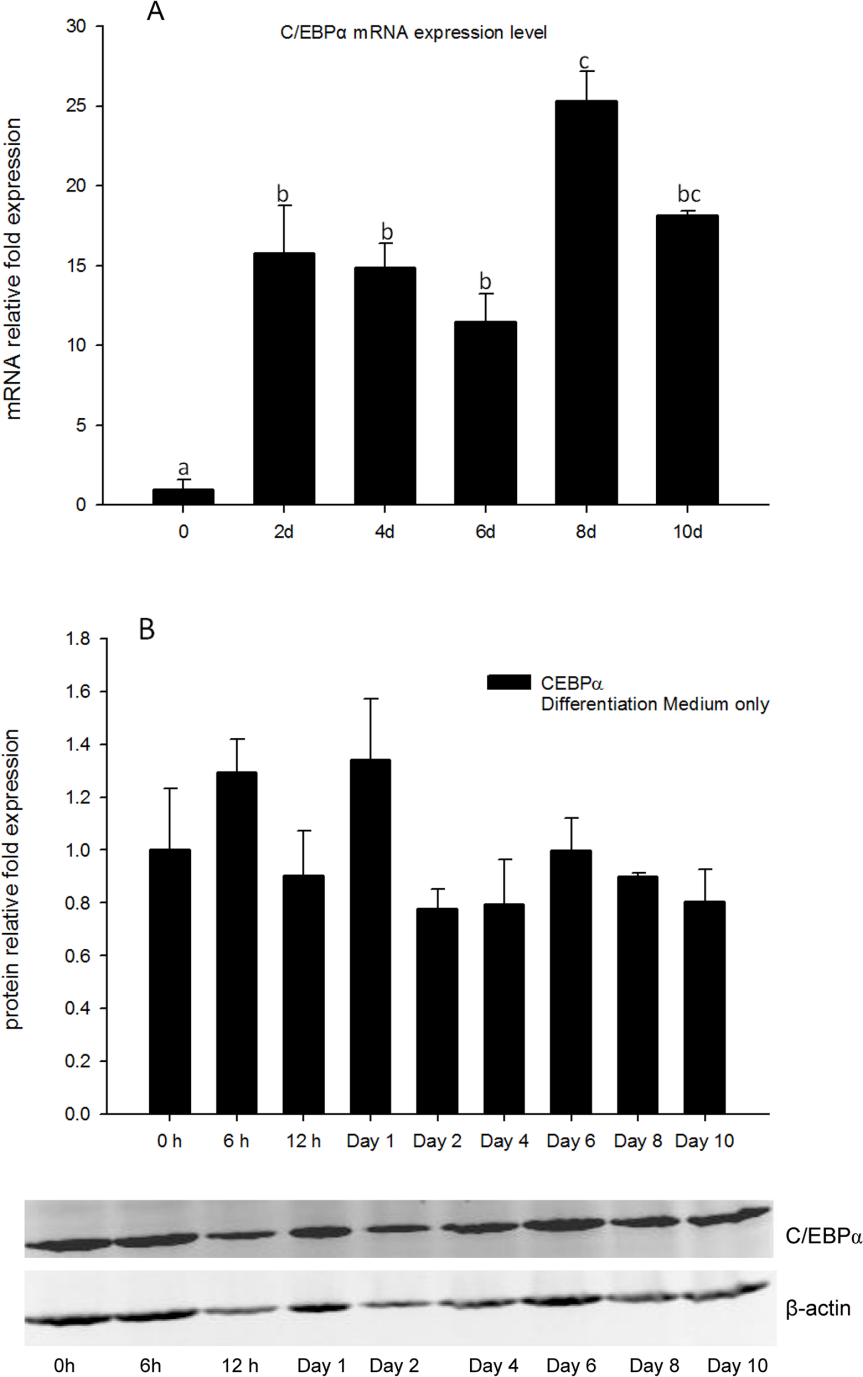
(A) Real-time PCR quantification of *C/EBPα* gene expression in DM treatment of 3T3-L1 cells on days 0, 2, 4, 6, 8, and 10 with *EEF2* used as endogenous control (∆Ct). Data were normalized to *C/EBPα* gene expression of the day 0 group (∆∆Ct). (B): Image showing Western blot analysis (Odyssey Dual Infrared Imaging System (Li-Cor)) of C/EBPα on 0, 6, and 12 h, days 1, 2, 4, 6, 8 and 10. β-actin was used as an internal protein loading control. Quantification of C/EBPα was normalized to β-actin. Data are means ± SE (n = 3). Different letters represent treatment effects that were significantly different (*P* < 0.05).

Both *C/EBPα* mRNA expression and protein level were measured in DM only treatments as a reference to help understand regulation of this gene in adipocyte differentiation (Fig 4). Gene expression of *C/EBPα* was increased from days 2 to 10, and reached the maximum at day 8 (Fig 4A), however, total cell protein levels of C/EBPα did not change significantly from 0 h to day 10 (Fig 4B), and appeared to remain at relatively high levels throughout the test period.

**Fig 5:**
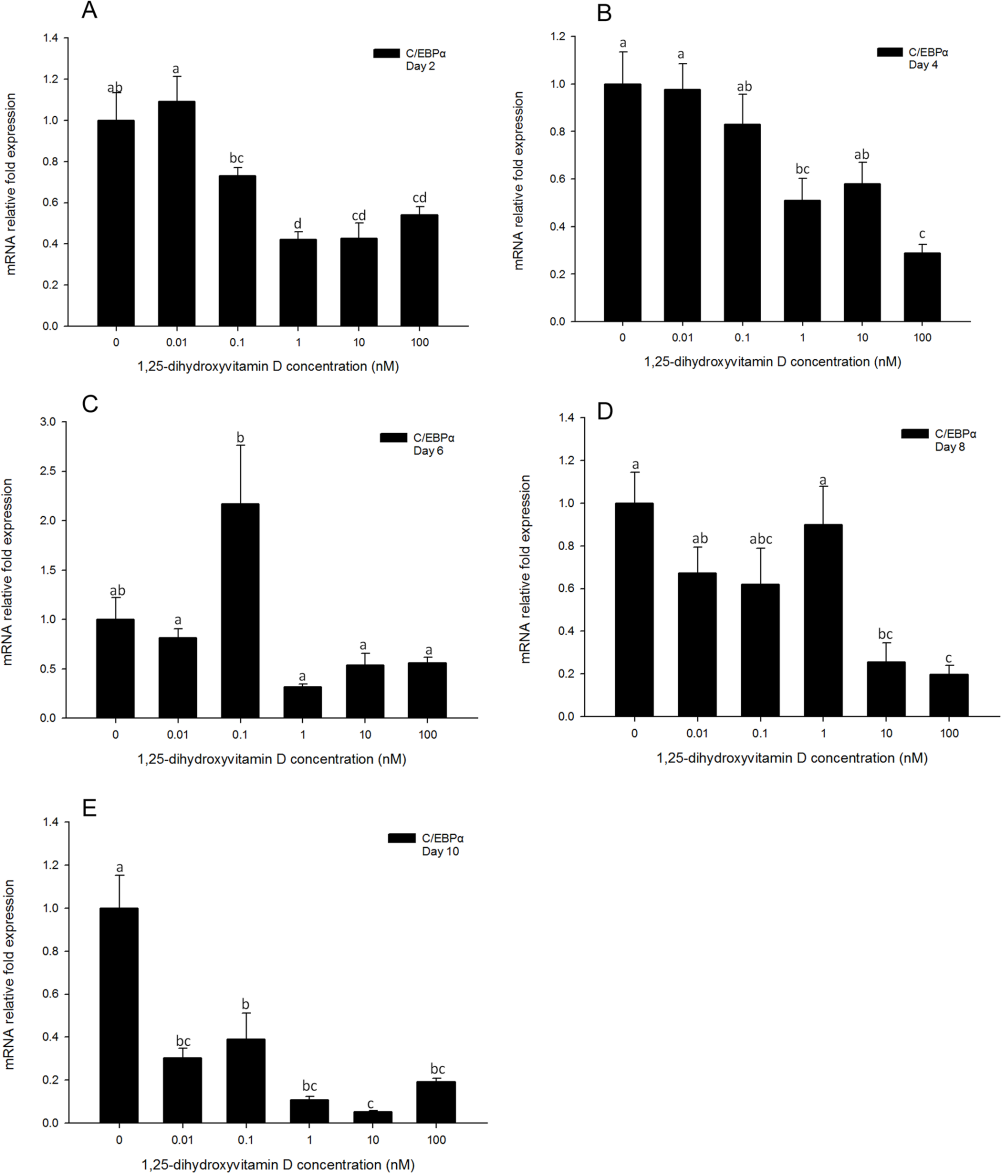
Real-time PCR quantification of *C/EBPα* gene expression in 3T3-L1 cells on days 2 (A), 4 (B), 6 (C), 8 (D) and 10 (E). Cells were treated with DM in the presence or absence of 0.01, 0.1, 1, 10, and 100 nM 1, 25 - (OH)_2_D_3_ and *EEF2* was used as endogenous control (∆Ct). Data were normalized to *C/EBPα* gene expression of positive control (DM) at the corresponding time point (∆∆Ct). Data are means ± SE (n = 3). Different letters represent treatment effects that were significantly different (*P* < 0.05).

Similarly to *PPARγ*, no significant changes in *C/EBPα* gene expression levels were observed in the cells treated with low concentrations (0.01 and 0.1 nM) of 1, 25 - (OH)_2_D_3_ as compared to the positive control cells at all time-points measured. (Fig 5). Cells treated with high concentrations (100, 10, and 1 nM) of 1, 25 - (OH)_2_D_3_ showed significant inhibition of *C/EBPα* expression as compared to the positive control for days 2 and 4 (Fig 5A and 5B). Similarly to *PPARγ* expression, *C/EBPα* gene expression levels showed no significant difference between treatments groups on day 6 (Fig 5C). The inhibitory efficacy of 1, 25 - (OH)_2_D_3_ was significant on days 8 and 10 in 1, 25 - (OH)_2_D_3_ treatment groups compared to DM only group (Fig 5D and 5E). This suggests that similarly to regulation of *PPARγ* expression, that 1, 25 - (OH)_2_D_3_ treatments had significant inhibitory effects on *C/EBPα* gene transcription, and this efficacy lasted until day 10.

Total cell protein levels of C/EBPα were also determined (S2 Fig). In the early time points (6 and 12 h), C/EBPα protein levels were not changed in the 1, 25 - (OH)_2_D_3_ treatment groups and DM only group compared to negative control (S2A and S2B Fig). Furthermore, there were no significant differences in C/EBPα protein levels compared to DM only group to 1, 25 - (OH)_2_D_3_ treated groups at all the time points, suggesting that total cellular C/EBPα protein levels were not influenced by 1, 25 - (OH)2D_3_ treatment.

### In the early time points, Vitamin D receptor gene expression is inhibited by high concentrations of 1, 25 - (OH)_2_D_3_

**Fig 6:**
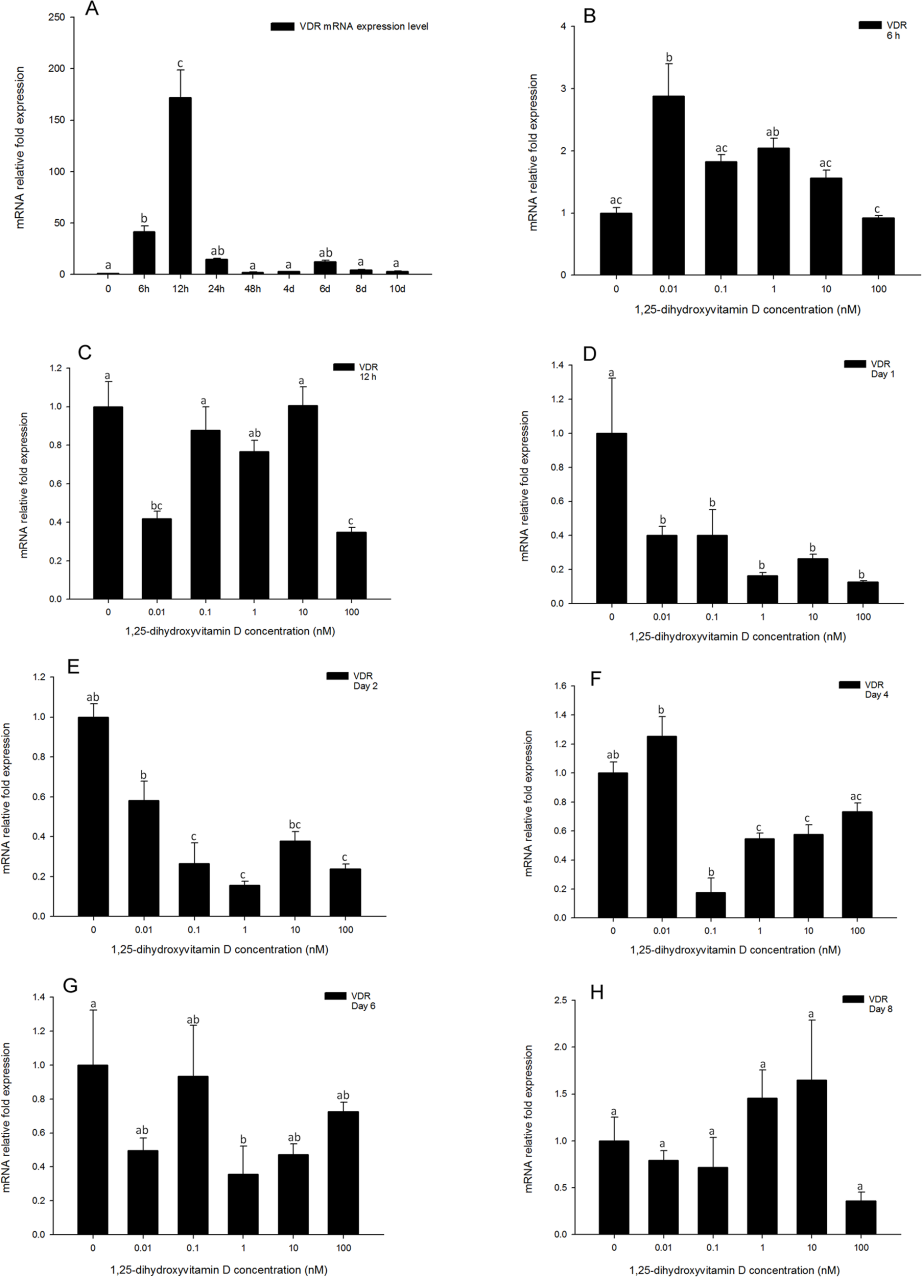
Real-time PCR quantification of *VDR* gene expression in 3T3-L1 cells (A): in the positive control treatment (DM) at 0, 6, and 12 h, and days 1, 2, 4, 6, 8, and 10. (B to I). Cells were treated with DM in the presence or absence of 0.01, 0.1, 1, 10, and 100 nM 1, 25 - (OH)_2_D_3_ and *EEF2* was used as endogenous control (∆Ct). Data were normalized to *VDR* gene expression of the positive control (DM) at the corresponding time point (∆∆Ct). (B) 6 h, (C) 12 h, (D) day 1, (E) day 2, (F) day 4 (G) day 6, (H) day 8 and (I) day 10. Data are means ± SE (n = 3). Different letters represent treatment effects that were significantly different (*P* < 0.05).

To better understand the expression pattern of vitamin D receptor, VDR mRNA expression levels were quantified from 0 h to day 10 in DM only treatments (Fig 6A). Gene expression of *VDR* increased from 6 h, reached a maximum at 12 h, and then decreased after 24 h. These results show that *VDR* was induced in the early time points of adipocyte differentiation, suggesting it may play an important role in inhibition of adipocyte differentiation by 1, 25 - (OH)_2_D_3_.

*Vitamin D receptor* gene expression was also determined in response to 1, 25 - (OH)_2_D_3_ treatments at 6 and 12 h, and on days 1, 2, 4, 6, 8, 10. At 6 and 12 h (Fig 6B and 6C), *VDR* gene expression was only inhibited in cells treated with the highest concentration (100 nM) of 1, 25 - (OH)_2_D_3_. Significant changes in *VDR* gene expression levels were observed in the cells treated with high concentrations (100, 10 and 1 nM) of 1, 25 - (OH)_2_D_3_ as compared to the positive control cells on days1, 2, and 4. (Fig 6D–6F). Cells treated with lower concentrations of 1, 25 - (OH)_2_D_3_ showed no significant inhibition of *VDR* expression as compared to DM only group at all the time points except day 10 (Fig 6B–6I). On day 10, *VDR* expression was inhibited by 1, 25 - (OH)_2_D_3_ treatments at all the concentrations (Fig 6I). This suggests that similarly to regulation of *PPARγ and C/EBPα* expression, 1, 25 - (OH)_2_D_3_ treatments had significant inhibitory effects on *VDR* gene transcription, especially early (days 1 and 2) in adipocyte differentiation. This provides robust evidence that 1, 25 - (OH)_2_D_3_ regulates VDR, and that the effect is consistent with a biological role for 1, 25 - (OH)_2_D_3_ in adipocyte differentiation.

### There is no effect of 1, 25-Dihydroxyvitamin D treatment on C/EBPβ gene expression levels

**Fig 7:**
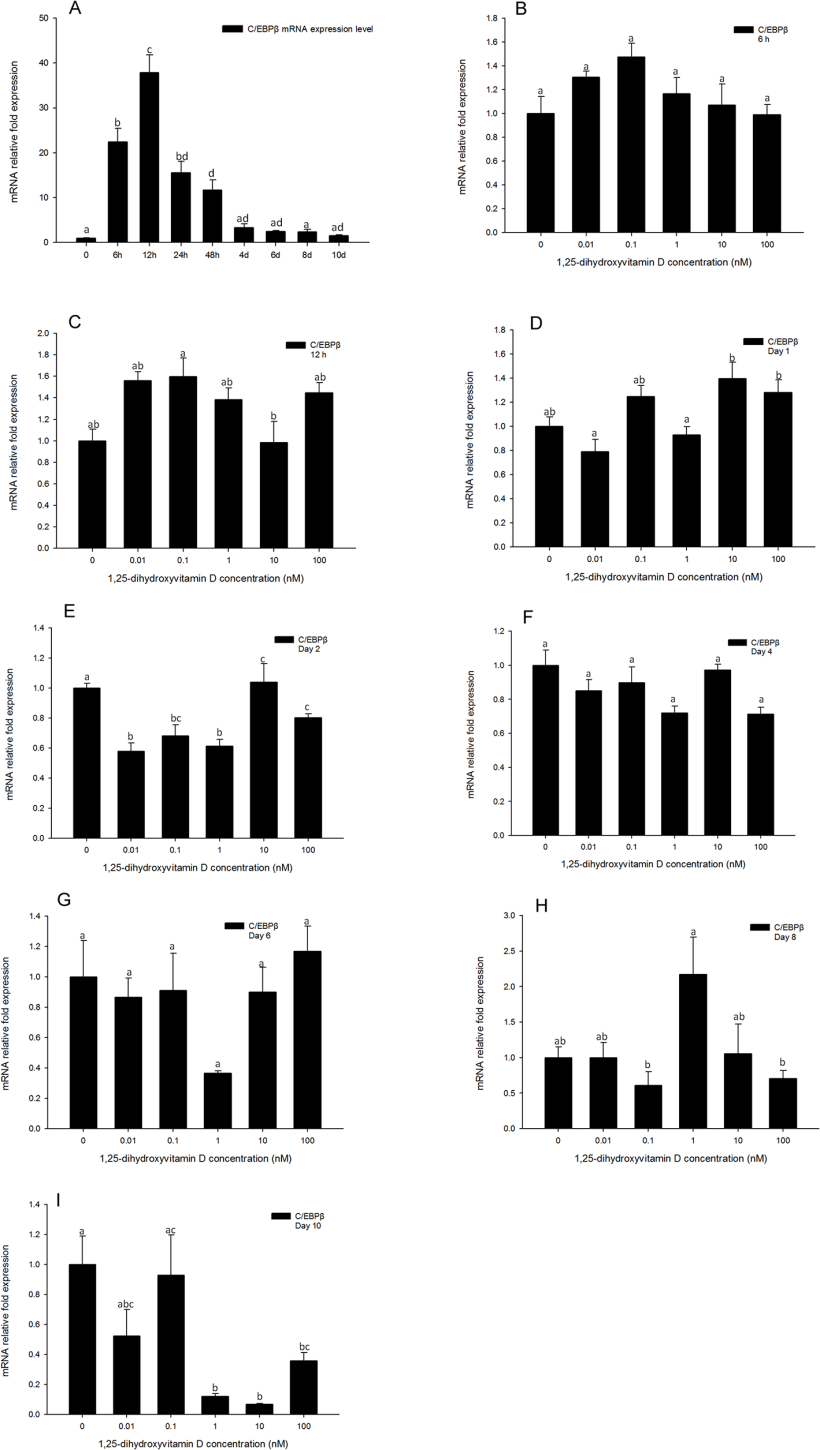
Real-time PCR quantification of *C/EBPβ* gene expression in 3T3-L1 cells (A): in the positive control treatment (DM) at 0, 6, and 12 h, and days 1, 2, 4, 6, 8, and 10. (B to I). Cells were treated with DM in the presence or absence of 0.01, 0.1, 1, 10, and 100 nM 1, 25 - (OH)_2_D_3_ and *EEF2* was used as endogenous control (∆Ct). Data were normalized to *C/EBPβ* gene expression of the positive control (DM) at the corresponding time point (∆∆Ct). (B) 6 h, (C) 12 h, (D) day 1, (E) day 2, (F) day 4 (G) day 6, (H) day 8 and (I) day 10. Data are means ± SE (n = 3). Different letters represent treatment effects that were significantly different (*P* < 0.05).

Gene expression levels of *C/EBPβ* in the positive control (Fig 7A) were increased after 6 h, and reached the highest expression level at 12 h, then decreased after day 2. Gene expression of *C/EBPβ* was determined at 6, 12, 24 h and days 2, 4, 6, 8 10 by real-time PCR. Unlike *PPARγ* and *C/EBPα*, *C/EBPβ* gene expression level was not impacted by 1, 25 - (OH)_2_D_3_ compared to DM only group at any time points tested, up to day 10 (Fig 7B–7H), suggesting that 1, 25 - (OH)_2_D_3_ has no effect on *C/EBPβ* gene expression levels. However, at day 10, the high concentrations (100, 10, and 1 nM) of 1, 25 - (OH)_2_D_3_ showed inhibitory effects on *C/EBPβ* gene expression (Fig 7I). On day 10, the expression level of *C/EBPβ* was very low (Fig 7A). Despite its low expression level, 1, 25 - (OH)_2_D_3_ had a suppressive effect on C/EBPβ expression at this time point.

### C/EBPδ gene expression was not changed in response to 1, 25 - (OH)_2_D_3_ treatments

**Fig 8:**
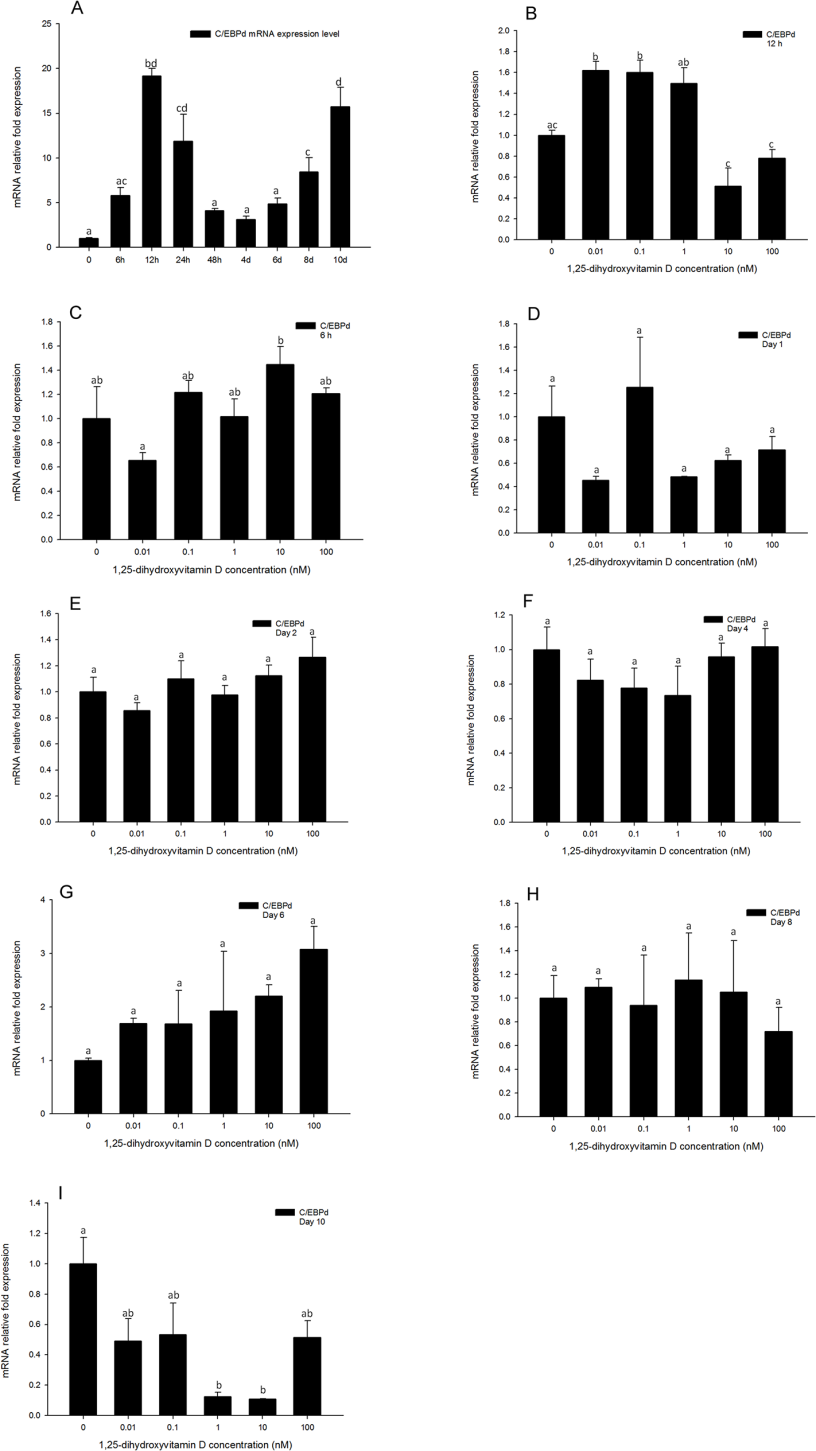
Real-time PCR quantification of *C/EBPδ* gene expression in 3T3-L1 cells (A): in the positive control treatment (DM) at 0, 6, and 12 h, and days 1, 2, 4, 6, 8, and 10. (B to I). Cells were treated with DM in the presence or absence of 0.01, 0.1, 1, 10, and 100 nM 1, 25 - (OH)_2_D_3_ and *EEF2* was used as endogenous control (∆Ct). Data were normalized to *C/EBPδ* gene expression of the positive control (DM) at the corresponding time point (∆∆Ct). (B) 6 h, (C) 12 h, (D) day 1, (E) day 2, (F) day 4 (G) day 6, (H) day 8 and (I) day 10. Data are means ± SE (n = 3). Different letters represent treatment effects that were significantly different (*P* < 0.05).

This member of the C/EBP family of transcription factors is induced in the early process of adipogenesis. Thus we quantified its gene expression levels at 6, 12, and 24 h, and continued to monitor its expression through days 2, 4, 6, 8, and 10. Similarly to *C/EBPβ*, *C/EBPδ* gene expression levels of 1, 25 - (OH)_2_D_3_ treated cells were generally not inhibited compared to the DM only treated group (Fig 8B–8I), even at the highest concentration of 1, 25 - (OH)_2_D_3_. This suggests that the inhibitory efficacy of 1, 25 - (OH)_2_D_3_ in adipogenesis does not impact the expression of *C/EBPδ*. Analysis of C/*EBPδ* gene expression in the positive control during adipocyte differentiation indicated that it is increased after 6 h, reaching the highest point at 12 h, and then decreases after 24 h (Fig 8A). Interestingly, the expression level of *C/EBPδ* was again increased after day 8, and reached a similar high expression level compared to 12 h on day 10 (Fig 8A). This suggests that the *C/EBPδ* gene may not only be induced and have a role in the early process of adipogenesis, but may also have a role in the latter stages of adipogenesis.

### Gene expression of FABP4 is highly responsive to 1, 25 - (OH)_2_D_3_ treatments

**Fig 9:**
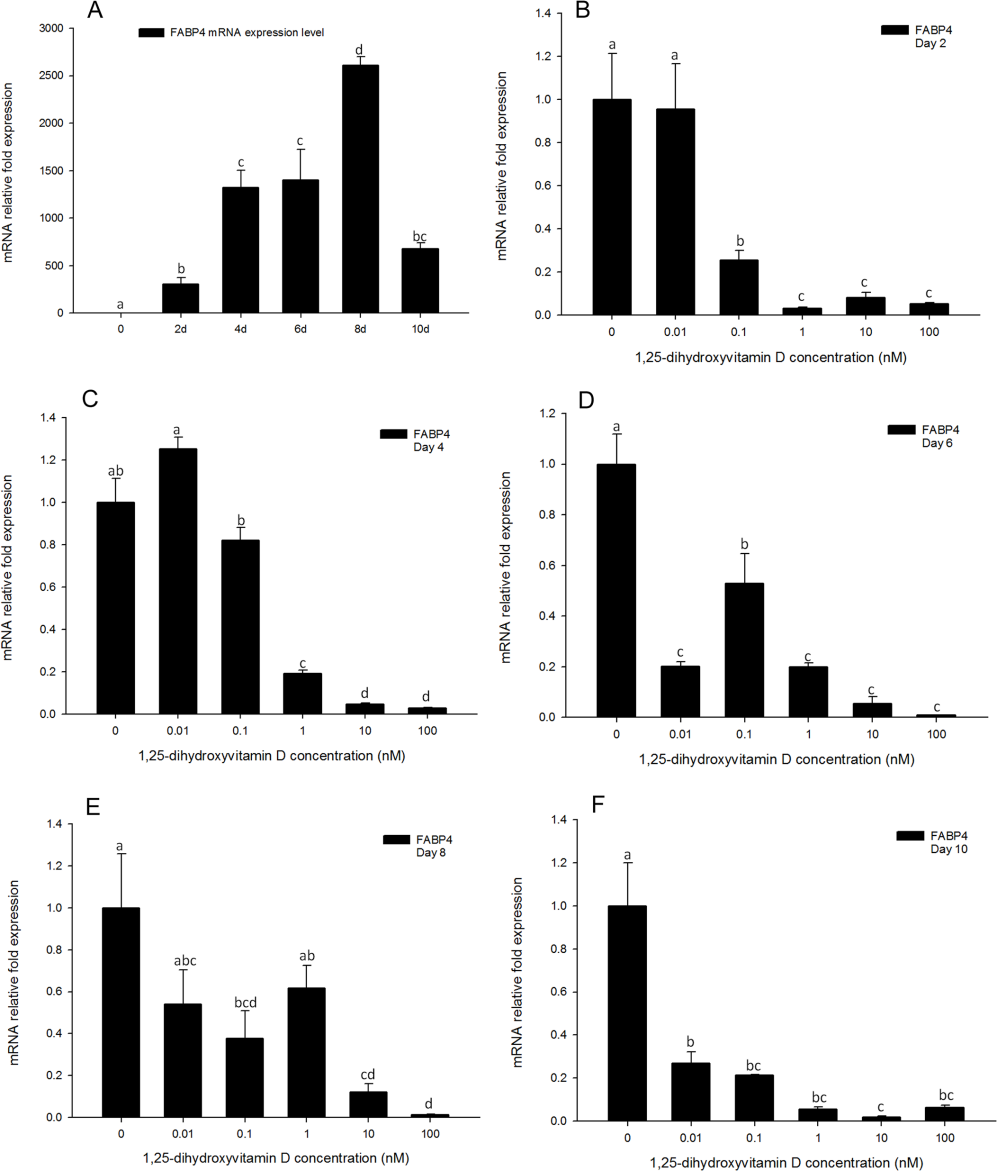
Real-time PCR quantification of *FABP4* gene expression in 3T3-L1 cells (A): in the positive control treatment (DM) on days 0, 1, 2, 4, 6, 8, and 10 (B to F). Cells were treated with DM in the presence or absence of 0.01, 0.1, 1, 10, and 100 nM 1, 25 - (OH)_2_D_3_ and *EEF2* was used as endogenous control (∆Ct). Data were normalized to *FABP4* gene expression of the positive control (DM) at the corresponding time point (∆∆Ct). (B) day 2, (C) day 4 (D) day 6, (E) day 8 and (F) day 10. Data are means ± SE (n = 3). Different letters represent treatment effects that were significantly different (*P* < 0.05).

In the positive control treatments, the expression pattern of *FABP4* was similar to that of *PPARγ*, increasing after day 2, and reaching the highest expression levels on day 8 but declining to similar levels to day 6 by day 10 (Fig 9F). Gene expression of *FABP4* was strongly inhibited by high concentrations (100, 10, and 1 nM) of 1, 25 - (OH)_2_D_3_ treatments at all the time points (Fig 9). Moreover, unlike *PPARγ* and *C/EBPα* expression levels, *FABP4* gene expression levels in response to 0.1 nM 1, 25 - (OH)_2_D_3_ were also significantly inhibited compared to the positive control at all time-points (Fig 9). The lowest concentration of 1, 25 - (OH)_2_D_3_ (0.01nM) treatments had no effect on *FABP4* gene expression on days 2 and 4 compared to the DM only group (Fig 9B and 9C). However, on day 6, even the lowest concentration of 1, 25 - (OH)_2_D_3_ showed an inhibitory effect on *FABP4* gene expression (Fig 9D). These effects were attenuated on days 8 and 10 (Fig 9E and 9F).

### Patterns of SREBP-1c expression resembled those of C/EBPβ and C/EBPδ expression, but was fleetingly inhibited on day 2

**Fig 10:**
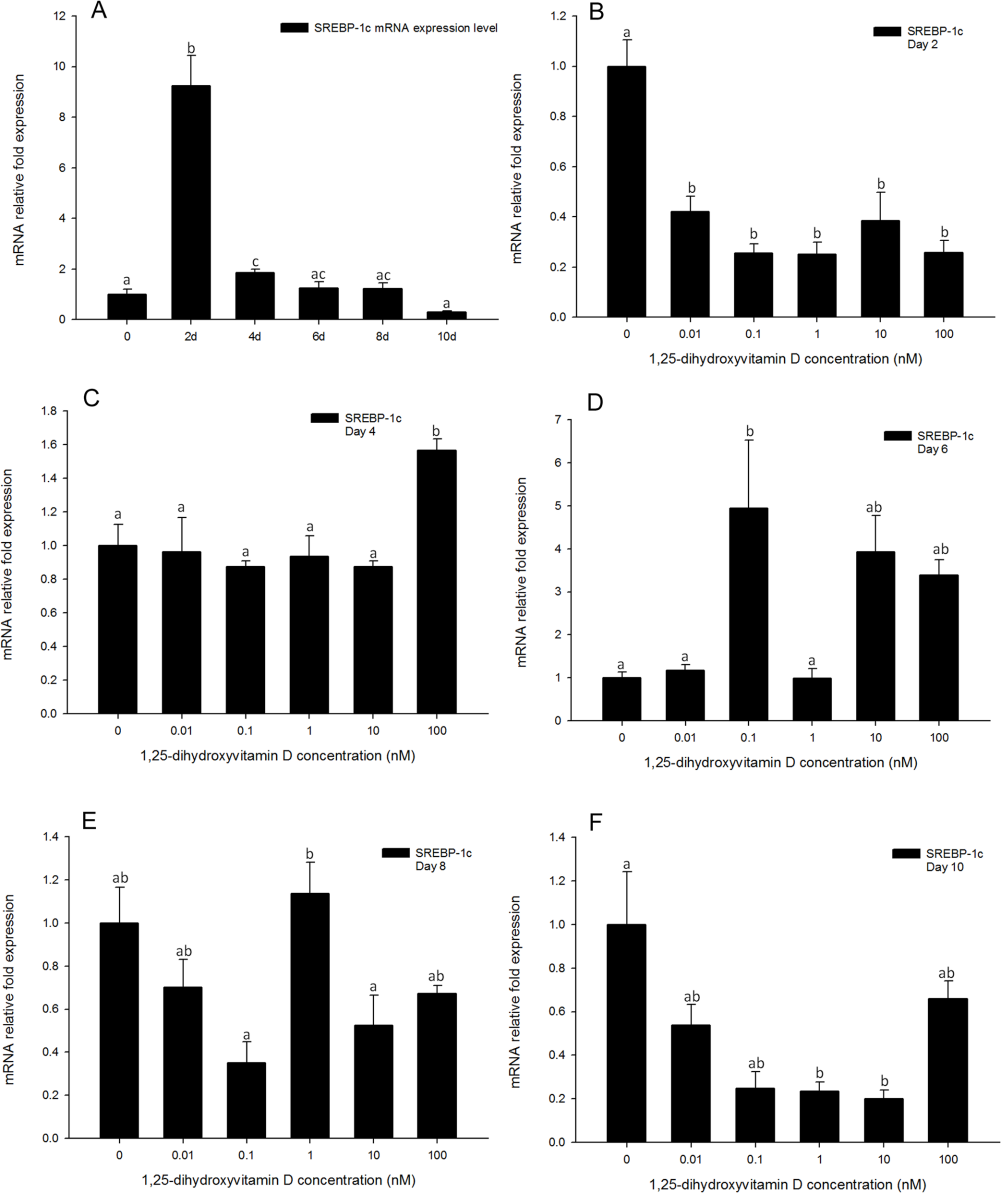
Real-time PCR quantification of *SREBP-1c* gene expression in 3T3-L1 cells (A): in the positive control treatment (DM) on days 0, 1, 2, 4, 6, 8, and 10 (B to F). Cells were treated with DM in the presence or absence of 0.01, 0.1, 1, 10, and 100 nM 1, 25 - (OH)_2_D_3_ and *EEF2* was used as endogenous control (∆Ct). Data were normalized to *SREBP-1c* gene expression of the positive control (DM) at the corresponding time point (∆∆Ct). (B) day 2, (C) day 4 (D) day 6, (E) day 8 and (F) day 10. Data are means ± SE (n = 3). Different letters represent treatment effects that were significantly different (*P* < 0.05).

The expression pattern of *SREBP-1c* in adipocyte differentiation showed that it was induced to the maximum expression level on day 2, and then decreased quickly from days 4 to 10 (Fig 10A). Interestingly, on day 2, cells treated with 1, 25 - (OH)_2_D_3_ (all concentrations tested, 100, 10, 1, 0.1 and 0.01 nM) showed significant inhibition of *SREBP-1c* gene expression as compared to the positive control (Fig 10B). However, this effect was rapidly attenuated. Similarly to *C/EBPβ* and *C/EBPδ*, *SREBP-1c* gene expression levels of 1, 25 - (OH)_2_D_3_ treated cells were generally not different from the positive control from days 4 to 10 (Fig 10C–10F). The inhibitory effect of 1, 25 - (OH)_2_D_3_ on day 2 coincides with the time point of maximum *SREBP-1c* expression (Fig 10A), suggesting that 1, 25 - (OH)_2_D_3_ only has an effect when *SREBP-1c* reached a high expression level. These results suggest that *SREBP-1c* may also play an important role in the 1, 25 - (OH)_2_D_3_ modulation pathway, and may have interaction with 1, 25 - (OH)_2_D_3_ during the early stages of adipogenesis.

### The inhibitory effect of 1, 25 - (OH)_2_D_3_ on SCD-1 gene expression levels was more gradual compared to PPARγ, C/EBPα or FABP4 expression

**Fig 11:**
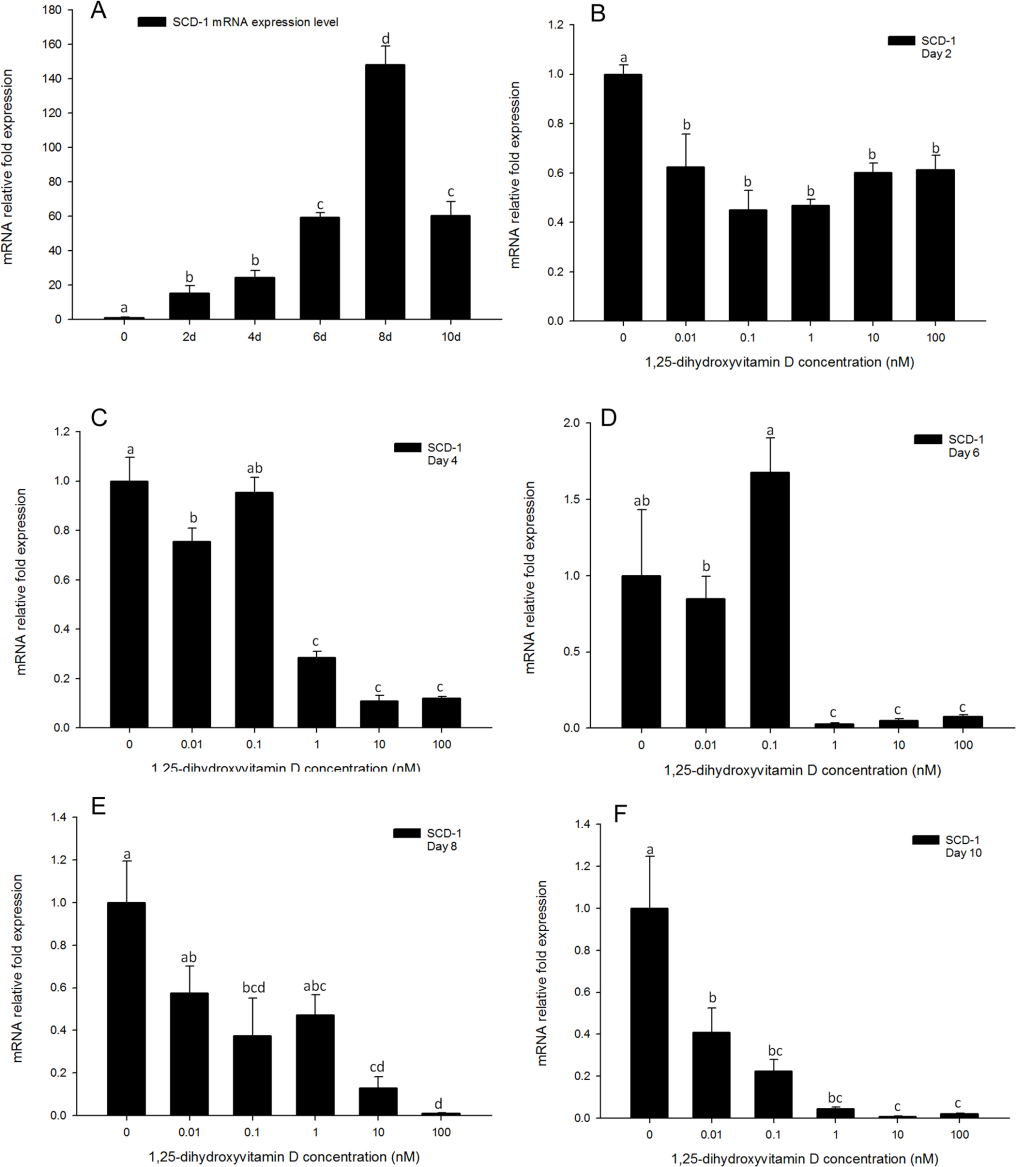
Real-time PCR quantification of *SCD-1* gene expression in 3T3-L1 cells (A): in the positive control treatment (DM) on days 0, 1, 2, 4, 6, 8, and 10 (B to F). Cells were treated with DM in the presence or absence of 0.01, 0.1, 1, 10, and 100 nM 1, 25 - (OH)_2_D_3_ and *EEF2* was used as endogenous control (∆Ct). Data were normalized to *SCD-1* gene expression of the positive control (DM) at the corresponding time point (∆∆Ct). (B) day 2, (C) day 4 (D) day 6, (E) day 8 and (F) day 10. Data are means ± SE (n = 3). Different letters represent treatment effects that were significantly different (*P* < 0.05).

For the positive control, the expression pattern of *SCD-1* in adipocyte differentiation process was similar to that of *PPARγ* and *C/EBPα*. Expression of *SCD-1* was increased on day 2, and reached a maximum expression level on day 8, remaining relatively high on day 10 (Fig 11A). The inhibition of *SCD-1* gene expression was induced by all concentrations of 1, 25 - (OH)_2_D_3_ on day 2 (Fig 11B). However, its inhibitory effect at latter time points was more pronounced (Fig 11C–11F). Expression of *SCD-1* was inhibited by high concentrations (100, 10 and 1 nM) of 1, 25 - (OH)_2_D_3_ on day 4 (Fig 11C), showing a 70% inhibition effect at this time point. This continued to day 6 (Fig 11D), and reached a inhibition effect greater than 90% of the positive control, in all of the three high concentrations of 1, 25 - (OH)_2_D_3_ treatments. The efficacy of 1, 25 - (OH)_2_D_3_ was stronger after day 6, and all the five concentrations of 1, 25 - (OH)_2_D_3_ showed significant inhibition on days 8 and 10 (Fig 11E and 11F). This suggests *SCD-1* is strongly responsive to 1, 25 - (OH)_2_D_3,_ and may play an important role in the pathway of 1, 25 - (OH)_2_D_3_ regulation of adipogenesis.

### Gene expression of Pref-1 was altered in early time-points in response to high concentrations of 1, 25 - (OH)_2_D_3_

**Fig 12:**
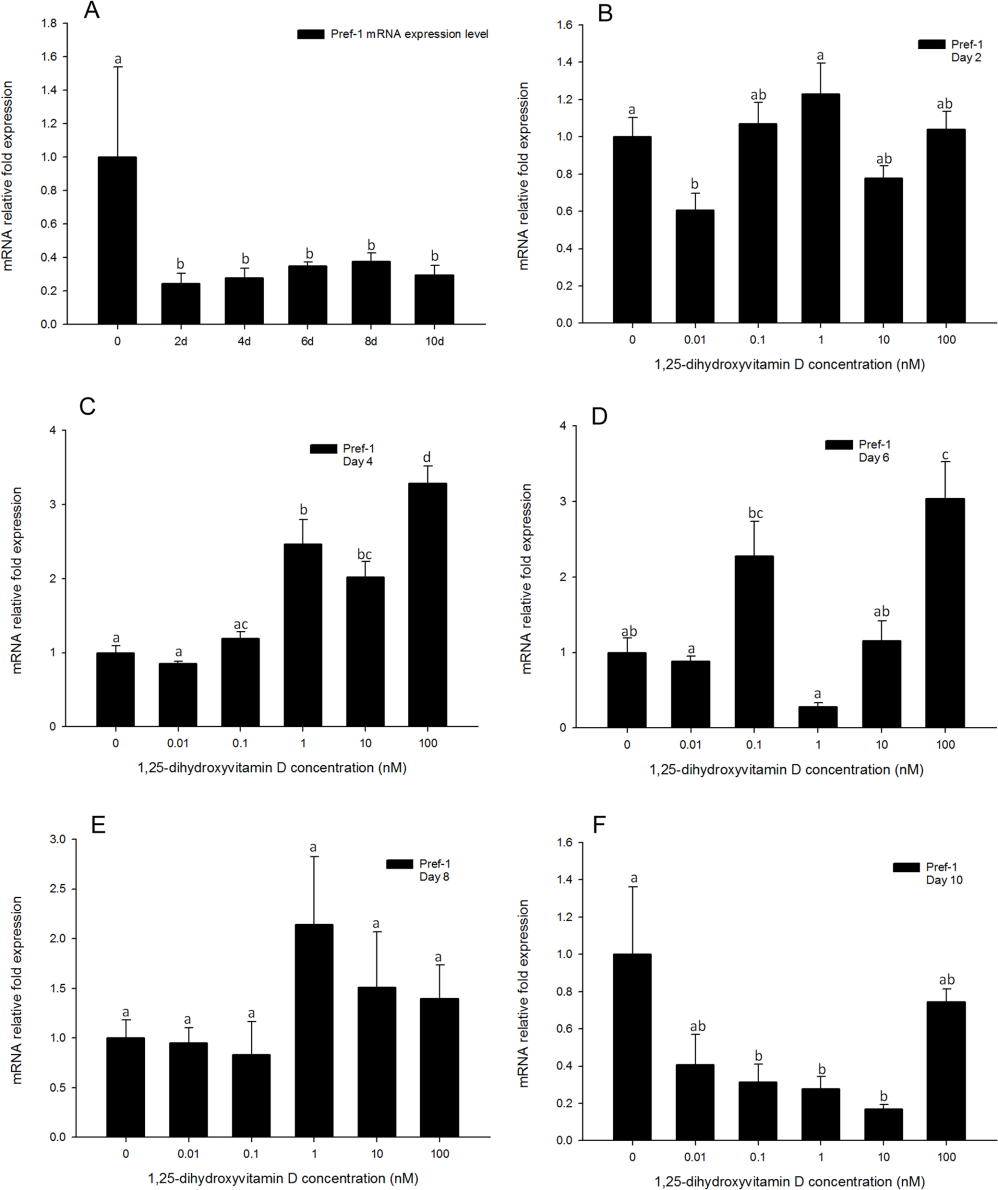
Real-time PCR quantification of *Pref-1* gene expression in 3T3-L1 cells (A): in the positive control treatment (DM) on days 0, 1, 2, 4, 6, 8, and 10 (B to F). Cells were treated with DM in the presence or absence of 0.01, 0.1, 1, 10, and 100 nM 1, 25 - (OH)_2_D_3_ and *EEF2* was used as endogenous control (∆Ct). Data were normalized to *Pref-1* gene expression of the positive control (DM) at the corresponding time point (∆∆Ct). (B) day 2, (C) day 4 (D) day 6, (E) day 8 and (F) day 10. Data are means ± SE (n = 3). Different letters represent treatment effects that were significantly different (*P* < 0.05).

In the positive control, the expression pattern of *Pref-1* as expected was decreased by day 2, and remained so through until day 10 (Fig 12A). Expression levels of *Pref-1* were not altered in any of the 1, 25 - (OH)_2_D_3_ treated cells on day 2 (Fig 12B), values being similar to the positive control. Cells treated with high concentrations of 1, 25 - (OH)_2_D_3_ (100, 10, and 1 nM) showed a significant increase in *Pref-1* gene expression levels from days 4 to 6 (Fig 12C and 12D), suggesting greater retention of the preadipocyte phenotype. By day 8, all effects from 1, 25 - (OH)_2_D_3_ treatments appeared to be attenuated, although the inhibitory effect was at least partially regenerated on day 10 (Fig 12E and 12F). This results suggest that *Pref-1* expression responds to 1, 25 - (OH)_2_D_3_ in the latter stages of adipocyte differentiation, and may also plays a role in the pathways of 1, 25 - (OH)_2_D_3_ inhibited adipogenesis.

### Relative luciferase activity of C/EBPα promoter activity was not affected by 1, 25 - (OH)_2_D_3_ treatment

**Fig 13:**
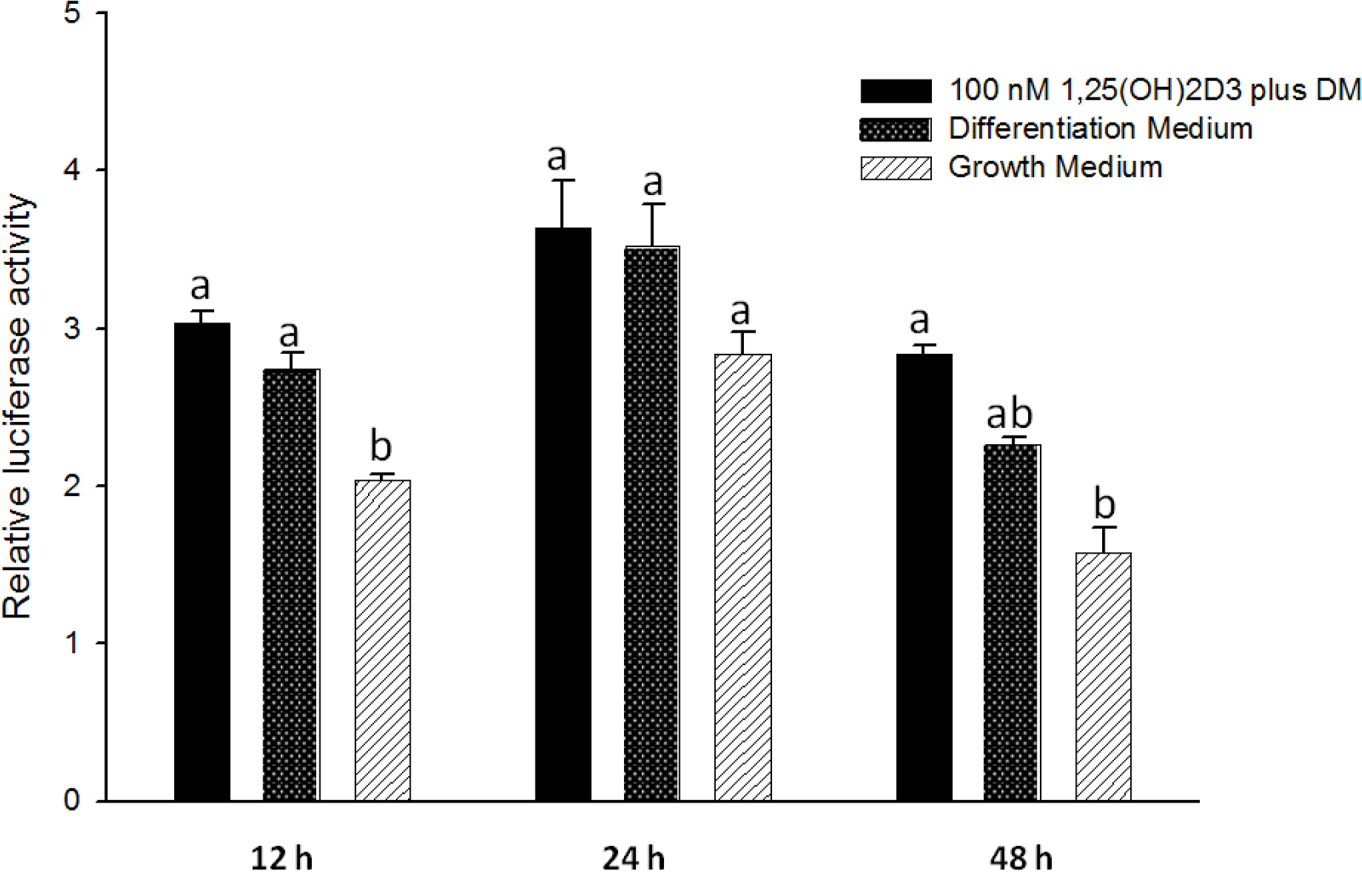
Mouse 3T3-L1 cells were transfected with pGL4.10 (luc2/-500*CEBPa*) in triplicate. Following incubation with differentiation medium only, growth medium only, or differentiation medium with 1, 25(OH)_2_D_3_ (100nM). Fire-fly and Renilla luciferase activity units were measured at 0, 12, 24 and 48 h. The firefly luciferase activity units were normalized to Renilla luciferase activity units. Data are normalized as fold activation relative to 0 h and shown as means ± SE (n = 3). Different letters represent treatment effects that were significantly different (*P* < 0.05) within each time-point.

This study was conducted to investigate *C/EBPα* promoter activity in response to transient exposure of cells for 0, 12, 24 and 48 h to 100 nM 1, 25 - (OH)_2_D_3_ plus differentiation medium, differentiation medium only, and growth medium only. The data obtained with 1, 25 - (OH)_2_D_3_ treatment indicated no change *C/EBPα* promoter activity at any time points, 12, 24 and 48 h, compared to differentiation medium, suggesting no effects of 1, 25 - (OH)_2_D_3_ on *C/EBPα* promoter activity (Fig 13). The promoter activities of *C/EBPα* from cells treated with both differentiation medium and differentiation medium plus 1, 25 - (OH)_2_D_3_ were significantly higher than growth medium alone (Fig 13), suggesting that the *C/EBPα* promoter is stimulated within the first 48 h of adipocyte differentiation.

## Discussion

Although the inhibitory effect of 1, 25 - (OH)_2_D_3_ in adipogenesis has been reported for more than a decade, the molecular mechanisms underlying this inhibition remains unclear. To explore this important question, we have performed a systematic investigation aimed at studying the molecular events during the adipocyte differentiation response to 1, 25 - (OH)_2_D_3_. The 3T3-L1 cell line is a major model used in developing understanding of adipocyte differentiation and key adipogenic gene expression. Our strategy was to take advantage of this well-defined adipogenic model and identify the molecular changes at each stage that resulted from 1, 25 - (OH)_2_D_3_ treatments. We report here that lipid accumulation and expression levels of adipogenic specific genes were inhibited in vitro by high concentrations (1, 10 and 100 nM) of 1, 25 - (OH)_2_D_3_ but not by lower concentrations (0.1 and 0.01 nM). As discussed in greater detail below, lipid accumulation was inhibited by the high concentrations of 1, 25 - (OH)_2_D_3_, at levels comparable to the negative control, by day 10. The lower concentrations of 1, 25 - (OH)_2_D_3_ have slight inhibitory effects on lipid accumulation compared to the positive control. Gene expression levels of *PPARγ*, *C/EBPα*, *VDR*, *FABP4* and *SCD-1* were inhibited by the high concentrations of 1, 25 - (OH)_2_D_3_ throughout the experimental period to day 10. However, the lower concentrations of 1, 25 - (OH)_2_D_3_ had no inhibitory effect. Gene expression levels of *C/EBPβ* and *C/EBPδ* were not affected by 1, 25 - (OH)_2_D_3_ treatments, at any of the concentrations tested. We also studied the effects of 1, 25 - (OH)_2_D_3_ on *C/EBPα* promoter activity. There appeared to be no inhibitory effect of 1, 25 - (OH)_2_D_3_ on the activity of the *C/EBPα* promoter. The present study has also provided a detailed temporal analysis of key adipogenic gene expression across time points from days 0 to 10 during the adipocyte differentiation process. These data demonstrate at least three important observations: 1) high concentrations of 1, 25 - (OH)_2_D_3_ have strongly inhibitory effects on adipogenesis, and this effects persist through day10, 2) not all of the key adipogenic genes (e.g. *C /EBPβ* and *C/EBPδ*) interact with 1, 25 - (OH)_2_D_3_, and 3) the pathway of 1, 25 - (OH)_2_D_3_ mediated inhibition of adipogenesis does not appear to involve the *C/EBPα* promoter.

1, 25-Dihydroxyvitamin D_3_ is an endocrine hormone that plays multiple physiological roles[21]. This secosteroid hormone is known to be critical for immune system function [22] and calcium and phosphate homeostasis[23,24]. 1, 25-Dihydroxyvitamin D_3_ is also known to affect adipocyte differentiation and metabolism [25]. 1, 25-Dihydroxyvitamin D_3_ is also the ligand of VDR, hence, VDR may play an important part in the inhibitory pathway of 1, 25 - (OH)_2_D_3_ in adipogenesis. The VDR has previously been reported to play an important role in the vitamin D signaling pathway in health and disease[25]. Kong and Li [26] found that VDR protein expression was very low in mouse 3T3-L1 preadipocytes, however, VDR expression increased dramatically by 4 h following treatment with adipogenic differentiation medium, and returned to baseline levels by day 2. 1, 25-Dihydroxyvitamin D_3_ treatment was able to stabilize VDR expression for at least another day. The mechanism of VDR stabilization by 1, 25 - (OH)_2_D_3_ is currently not known. However, the observation of VDR expression in the early time points of adipogenesis may provide a short window for 1, 25 - (OH)_2_D_3_ to inhibit adipogenesis[13]. The role of the VDR in pre-adipocyte differentiation in 3T3-L1 cells was also studied by Blumberg et al[15]. Their studies reported that the mRNA levels of VDR increased to a maximum by 6 h following initiation of adipocyte differentiation, and the protein levels of VDR reached a maximum by 12 h in the nucleus, and then declined to baseline level by day 2. These similar reports suggest that the inhibition of adipogenesis by 1, 25 - (OH)_2_D_3_ binding VDR may occur in the early time points (before day 2) during adipogenesis, however, the specific mechanism still remains unknown. In our studies, *VDR* gene expression pattern in adipocyte differentiation was measured from 0 h to day 10. The expression of *VDR* was induced after adipocyte differentiation was initiated (6 h), and reached the maximum expression level at 12 h, then declined after 24 h, which is consistent with previous literature reports. In addition, *VDR* gene expression was inhibited by 1, 25 - (OH)_2_D_3_ treatments at early time points. We report the novel finding that the interaction between 1, 25 - (OH)_2_D_3_ and its receptor leads to inhibition of down-stream expression of adipogenic-specific genes. This suggests that *VDR* plays an important role in the inhibitory pathway of 1, 25 - (OH)_2_D_3_ regulating adipocyte differentiation.

The C/EBP family is a class of basic-leucine zipper transcription factors, and does not form homo-or hetero dimers. Furthermore, their tissue distribution is not limited to adipose tissue[27]. The gene expression of several *C/EBP* family members is known to be regulated during adipogenesis, and they have been shown to be regulators of adipocyte differentiation. Both *C/EBPβ* and *C/EBPδ* mRNA and protein levels were reported to rise early and transiently in preadipocytes which have been induced to differentiate [28–30]. In the present study, real-time PCR results confirmed that during adipogenesis, *C/EBPβ* mRNA expression levels began to rise by 6 h, and reached a maximum by 12 h following induction, then declined to baseline level after 24 h (Fig 7A). The mRNA expression levels of *C/EBPδ* also increased by 6 h, and reached the highest level at 12 h, then decreased after 24 h (Fig 8A). These results are consistent with previous reports, and the timing of expression of these two genes was similar to VDR, suggesting that there may be an interaction between 1, 25 - (OH)_2_D_3_ through VDR binding, inhibiting adipogenesis and *C/EBPβ* or *C/EBPδ* expression during early adipogenesis. Blumberg et al [15] reported that 1, 25 - (OH)_2_D_3_ treatment inhibited *C/EBPβ* mRNA expression level, however, *C/EBPδ* gene expression did not change in response to 1, 25 - (OH)_2_D_3_. Their studies indicate that after binding with VDR, 1, 25 - (OH)_2_D_3_ inhibits adipogenesis via inhibiting *C/EBPβ* gene expression but not *C/EBPδ*. In contrast, Kong and Li [26] reported that 1, 25 - (OH)_2_D_3_ treatments did not influence the gene expression of either *C/EBPβ* or *C/EBPδ* [27]. In the present study, we quantified gene expression levels of both *C/EBPβ* and *C/EBPδ* in response to 1, 25 - (OH)_2_D_3_ treatment, and our results are similar to those reported by Kong and Li[26]. From 6 h to 24 h, and days 2 to 10, gene expression levels of *C/EBPβ* and *C/EBP*δ were not changed by 1, 25 - (OH)_2_D_3_ treatments. These data demonstrate that even though the gene expression of these C/EBP family members is stimulated in early adipogenesis, corresponding to the maximum expression time of VDR, these two factors are not included in the pathway of 1, 25 - (OH)_2_D_3_ inhibited adipogenesis.

The *PPAR* family is a group of transcriptional factors belonging to the nuclear hormone receptor superfamily. These transcriptional factors heterodimerize with another nuclear hormone receptor, retinoid X receptor (RXR), bind to the response elements of target gene promoters and function as active transcriptional factors[31]. When PPARs are heterodimerized with RXR, the complex is activated and transported to the nucleus to bind to specific sequences in promoter regions (termed as *PPAR* response elements, PPREs) of downstream target genes, activating their transcription[6,32]. There are three major isoforms: *PPARα*, *PPARδ*, and *PPARγ* [28]. The three isoforms have specific roles in lipid metabolism. Importantly, *PPARγ* plays an important role in triglyceride synthesis and adipocyte differentiation[32]. Activation of *PPARγ* expression occurs downstream of *C/EBPβ* and *C/EBPδ* transcription during the cascade of adipogenesis, and upstream of *C/EBPα*. In the present study, gene expression of *PPARγ* was highly inhibited by 1, 25 - (OH)_2_D_3_, from day 2 until day 10 (Fig 3). Moreover, the cellular response of *C/EBPα* to 1, 25 - (OH)_2_D_3_ was similar to that of *PPARγ*. The inhibition of 1, 25 - (OH)_2_D_3_ was persistent until day 10 (Fig 5). These data indicate that the 1, 25 - (OH)_2_D_3_ induced inhibition of adipogenesis in 3T3-L1 cells was associated with an inhibition of *PPARγ* and *C/EBPα* gene expression.

To confirm these results, the protein levels of both PPARγ and C/EBPα were measured using Western blot. Interestingly, the whole cell lysate from 1, 25 - (OH)_2_D_3_ plus DM treated cells had the highest PPARγ protein level from 6 h to day 4, and the whole cell lysate from DM only treated cells had lower PPARγ levels than the 1, 25 - (OH)_2_D_3_ treated cells, but comparable to growth medium treated cells. By day 6, the 1, 25 - (OH)_2_D_3_ plus DM treated cells and DM only treated cells had similar levels of PPARγ protein, however, by day 10, DM only treated cells had the highest PPARγ protein level, and PPARγ protein level from 1, 25 - (OH)_2_D_3_ plus DM treated cells was decreased to the same level as growth medium only treated cells (S1 Fig). Protein levels of C/EBPα in the whole cell lysate were not changed in response to 1, 25 - (OH)_2_D_3_ treatments on any of the time points in comparison to DM only treated cells (S2 Fig), suggesting that C/EBPα protein was not influenced by 1, 25 - (OH)_2_D_3_ treatments. In previous studies by Blumberg et al[15], they reported the protein level of PPARγ and C/EBPα was inhibited by 1, 25 - (OH)_2_D_3_ treatments. However, these authors used the nuclear extracts to measure the protein level of these two transcriptional factors. In the present study, we used the whole cell lysate to measure the protein levels. These observations together suggest that regulation of PPARγ effects are not directly mediated at transcriptional or translational levels. Rather, mediation occurs via regulation of PPARγ activation and transport to the nucleus. Thus, we hypothesize that 1, 25 - (OH)_2_D_3_ treatments block the trafficking of PPARγ from the cytoplasm to the nucleus. Thus, PPARγ protein is not transferred into nucleus preventing activation of downstream target genes in adipogenesis. In contrast, without 1, 25 - (OH)_2_D_3_ treatment, the PPARγ protein in the DM only treated cells was readily transported into the nucleus, and functioned as transcriptional factor, inducing the downstream genes (e.g. *C/EBPα*, *FABP4*). Therefore, the protein level of PPARγ in DM only treated cells was lower compared to 1, 25 - (OH)_2_D_3_ treated cells. The protein levels of PPARγ and C/EBPα in DM only treated cells were measured from 0, 6, and 12 h to days 1, 2, 4, 6, 8, and 10. The protein levels of PPARγ were consistent with mRNA expression levels quantified by real-time PCR. Interestingly, unlike PPARγ, the protein levels of C/EBPα were only slightly changed throughout the experimental time points. We hypothesize that this may be because the C/EBPα protein has longer half-life than PPARγ or is accumulated in the cytoplasm before adipogenesis is initiated. The activity of the *C/EBPα* promoter was also measured using the Dual Reporter Luciferase Assay System (Promega, Madison, WI). Relative luciferase activity data showed that the activity of *C/EBPα* promoter appeared to be unchanged in response to 1, 25 - (OH)_2_D_3_ treatments. These intriguing data demonstrate that 1, 25 - (OH)_2_D_3_ treatments inhibit adipogenesis via inhibiting *PPARγ* and *C/EBPα* gene expression, and that *PPARγ* may play a more important role in this pathway in comparison to *C/EBPα*. Further studies are needed to explore the mechanism of *PPARγ* interaction with 1, 25 - (OH)_2_D_3_ in its inhibition of adipogenesis.

In the present study, *SREBP-1c* gene expression was only inhibited by 1, 25 - (OH)_2_D_3_ treatments on day 2, coinciding with its maximum expression level in the positive control treatment. Both the high (100, 10, and 1 nM) and low (0.1 and 0.01 nM) concentrations of 1, 25 - (OH)_2_D_3_ inhibited *SREBP-1c* gene expression on day 2 (Fig 10B). However, from days 4 to 10, the inhibitory effects were ameliorated, and the expression of *SREBP-1c* was not changed in response to 1, 25 - (OH)_2_D_3_ treatments, at any of the concentrations tested (Fig 10C–10F). These data indicate that the inhibition of *SREBP-1c* gene expression by 1, 25 - (OH)_2_D_3_ treatment was transient and corresponded with the d 2 time point, in which its expression rose, 10-fold in the positive control in comparison to the time-point at d 0. Thus, it is not clear whether *SREBP-1c* may be involved in the 1, 25 - (OH)_2_D_3_ signaling pathway that inhibits adipogenesis, showing a similar gene expression profile to *C/EBPβ* and also reflecting the profile observed for *C/EBPδ* at time-points up to d 6. These three genes are upstream of *PPARγ* in the transcriptional activation of adipogenesis, hence, the inhibition of adipogenesis caused by 1, 25 - (OH)_2_D_3_ may be unrelated to mechanisms involving the transcriptional factors that are expressed in the early stages of adipogenesis.

In additional to *PPARγ* and *C/EBPα*, gene expression levels of *FABP4* and *SCD-1* were strongly inhibited by 1, 25 - (OH)_2_D_3_ treatments. Gene expression of *FABP4* was strongly inhibited by the high concentrations (100, 10 and 1 nM) of 1, 25 - (OH)_2_D_3_ treatments from days 2 to 4 (Fig 9B and 9C). Moreover, from days 6 to 10, all the concentrations of 1, 25 - (OH)_2_D_3_ had significant inhibitory effects on *FABP4* gene expression (Fig 9D–9F). The inhibitory effects of 1, 25 - (OH)_2_D_3_ treatments on *SCD-1* gene expression were gradual in comparison to effects on *FABP4* expression. Inhibition by high concentrations of 1, 25 - (OH)_2_D_3_ began by day 4 (Fig 10C), and remained until day 8 (Fig 11D and 11E). However, by day 10, gene expression of *SCD-1* was inhibited by all concentrations of 1, 25 - (OH)_2_D_3_ tested (Fig 11F), and comparable to effects on *FABP4*. In previous reports, *FABP4* has been shown to have a *PPARγ* response element (PPRE) in its promoter region and *PPARγ* regulates gene expression of *FABP4*[32]. In 1, 25 - (OH)_2_D_3_ treated cells, *PPARγ* expression was significantly inhibited, and this effect also appeared to cause a negative action on gene expression of *FABP4*. Similarly to *FABP4*, *SCD-1* also plays an important role in adipogenesis. Its functions include incorporation of double bonds in fatty acids and synthesis of long chain fatty acids in adipocytes[32]. In the present study, *SCD-1* expression was gradually increased from days 2 to 10 with DM treatment, and significantly inhibited by 1, 25 - (OH)_2_D_3_ treatments, suggesting that *SCD-1* may play a role in the pathway of 1, 25 - (OH)_2_D_3_ inhibited adipogenesis. Mechanisms of *FABP4* and *SCD-1* gene expression in response to 1, 25 - (OH)_2_D_3_ still need to be explored further.

Preadipocyte factor 1 is a marker protein of preadipocytes and is not expressed in mature adipocytes[33]. During initiation of adipogenesis, the gene expression of Pref-1 decreases and the expression of key adipogenic genes increases[34]. We hypothesized that gene expression of *Pref-1* would decrease in treatments with differentiation medium, and would remain at higher levels in treatments with 1, 25 - (OH)_2_D_3_ when compared to in DM treated cells. In the present study, *Pref-1* gene expression was significantly lower compared to that of day 4, DM only treated cells, and remained at low levels through day 10 (Fig 12A). In the cells treated with 1, 25 - (OH)_2_D_3_, *Pref-1* gene expression was significantly higher than in DM only treated cells by day 6 (Fig 12B–12F), and declined to similar levels to the DM only group from days 8 to 10 (Fig 12E and 12F). These data support our hypothesis, and in the 1, 25 - (OH)_2_D_3_ treatments where *PPARγ*, *C/EBPα*, *FABP4* and *SCD-1* gene expression levels were inhibited, the expression of *Pref-1* gene correspondingly remained significantly higher than in DM only treatments.

In conclusion, lipid accumulation and the expression of key adipogenic key genes, *PPARγ*, *C/EBPα*, *FABP4*, and *SCD-1* were significantly inhibited by 1, 25 - (OH)_2_D_3_ treatments until day 10. Gene expression of *SREBP-1c* was transiently inhibited by 1, 25 - (OH)_2_D_3_ on day 2, and then rebounded back to levels similar to the low levels observed in DM treatment by days 4, 6, 8, and 10. In contrast, *C/EBP β* and *C/EBP δ* expression were not changed in response to 1, 25 - (OH)_2_D_3_ treatments. Our study has demonstrated that 1, 25 - (OH)_2_D_3_ represses adipogenesis via inhibition of the expression of *PPARγ*, but not *C/EBP β* or *C/EBP δ*, and hence, the adipogenic-specific genes (*C/EBPα*, *FABP4*, and *SCD-1*) downstream of *PPARγ* during the transcriptional cascade of adipogenesis, were also inhibited. In future, studies are needed to explore the mechanisms by which 1, 25 - (OH)_2_D_3_ interacts with *PPARγ* and regulates adipogenesis.

## Supporting Information

S1 Fig Representative images showing Western blot analysis (Odyssey Dual Infrared Imaging System (Li-Cor)) of PPARγ on 6 h (A), 12 h (B), days 1 (C), 2 (D), 4 (E), 6 (F), 8 (G) and 10 (H). Cells were treated with differentiation medium in the presence or absence of 100 and 1 nM 1, 25 - (OH)_2_D_3_, and basal growth medium. β-actin was used as an internal protein loading control. Quantification of PPARγ normalized to β-actin. Comparisons are with blank within day. Data are means ± SE (n = 3).
(DOCX)

S2 Fig Representative images showing Western blot analysis (Odyssey Dual Infrared Imaging System (Li-Cor)) of C/EBPα on 6 h (A), 12 h (B), days 1 (C), 2 (D), 4 (E), 6 (F), 8 (G) and 10 (H). Cells were treated with differentiation medium in the presence or absence of 100 and 1 nM 1, 25 - (OH)_2_D_3_, and basal growth medium. β-actin was used as an internal protein loading control. Quantification of PPARγ normalized to β-actin. Comparisons are with blank within day. Data are means ± SE (n = 3).
(DOCX)
