# Correction: Regulation of Adipogenesis and Key Adipogenic Gene Expression by 1, 25-Dihydroxyvitamin D in 3T3-L1 Cells

**DOI:** 10.1371/journal.pone.0134199

**Published:** 2015-07-24

**Authors:** Shuhan Ji, Matthew E. Doumit, Rodney A. Hill

The following information is missing from the funding section: The Department of Animal and Veterinary Science, College of Agriculture and Life Sciences, University of Idaho.
