# Supplementary material for: Regulation of Adipogenesis and Key Adipogenic Gene Expression by 1, 25-Dihydroxyvitamin D in 3T3-L1 Cells
Source: PLoS One. 2015 Jun 1;10(6):e0126142. doi: 10.1371/journal.pone.0126142 (PMC4451075; doi:10.1371/journal.pone.0126142)

**SUPPLEMENTARY FIGURE CAPTIONS**

Fig S1: Western Blot quantification of PPARγ protein expression. Representative images showing Western blot analysis (Odyssey® Dual Infrared Imaging System (Li-Cor)) of PPARγ on 6 h (A), 12 h (B), days 1 (C), 2 (D), 4 (E), 6 (F), 8 (G) and 10 (H). Cells were treated with differentiation medium in the presence or absence of 100 and 1 nM 1, 25 - (OH)_2_D_3_, and basal growth medium. β-actin was used as an internal protein loading control. Quantification of PPARγ normalized to β-actin. Comparisons are with blank within day. Data are means ± SE (n = 3).


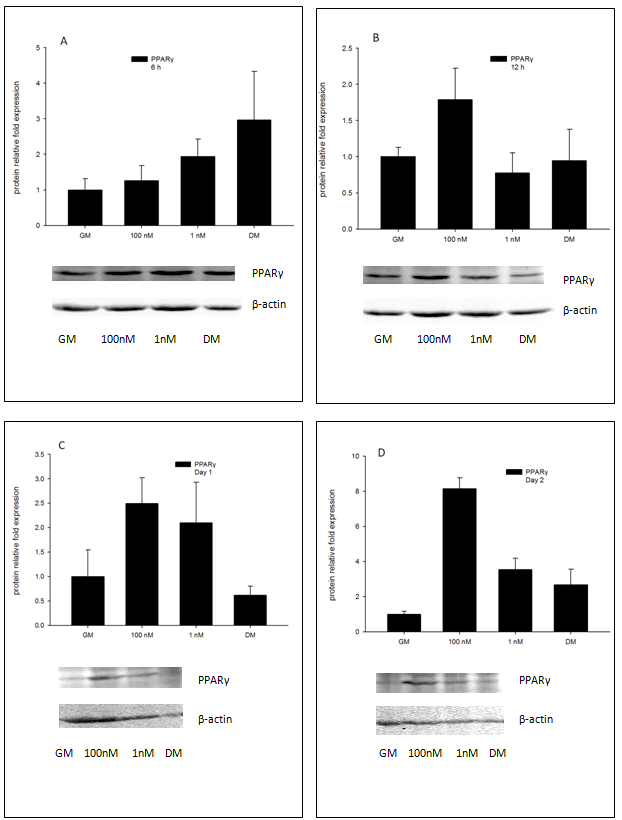


Fig S1 (Cont.): Western Blot quantification of PPARγ protein expression


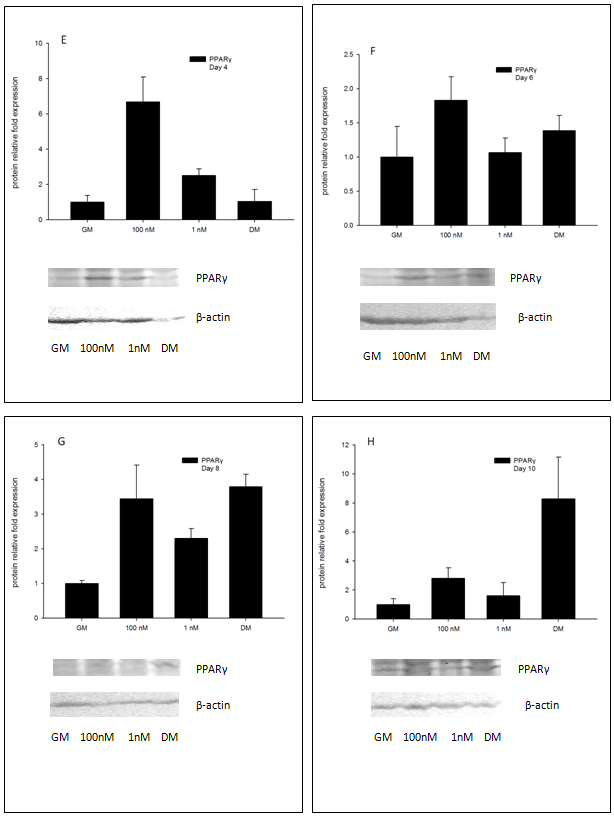

Supplement: S1 Fig — Cells were treated with differentiation medium in the presence or absence of 100 and 1 nM 1, 25 - (OH)2D3, and basal growth medium. β-actin was used as an internal protein loading control. Quantification of PPARγ normalized to β-actin. Comparisons are with blank within day. Data are means ± SE (n = 3). (DOCX) [file pone.0126142.s001.docx]
